# Update on the global epidemiology of intussusception: a systematic review of incidence rates, age distributions and case-fatality ratios among children aged <5 years, before the introduction of rotavirus vaccination

**DOI:** 10.1093/ije/dyz028

**Published:** 2019-03-16

**Authors:** Andrew D Clark, Mateusz Hasso-Agopsowicz, Matthew W Kraus, Lisa K Stockdale, Colin F B Sanderson, Umesh D Parashar, Jacqueline E Tate

**Affiliations:** 1 London School of Hygiene and Tropical Medicine, London, UK; 2 World Health Organization, Geneva, Switzerland; 3 Last Mile Health, Boston, USA; 4 University of Oxford, Oxford, UK; 5 Centers for Disease Control and Prevention, Atlanta, USA

**Keywords:** Intussusception, epidemiology, age, incidence, mortality

## Abstract

**Background:**

In some countries that have introduced oral rotavirus vaccines, a small but elevated risk of intussusception—a rare bowel disorder—has been reported. Updated estimates on the global epidemiology of intussusception are needed to help predict the potential number of intussusception cases that could be caused by the vaccine in different settings.

**Methods:**

We estimated incidence rates, age distributions and case-fatality ratios (CFRs) for intussusception hospital admissions among children aged <5 years, before the introduction of rotavirus vaccines. We included all articles identified in a systematic review between January 2002 and January 2018, and contacted authors for more granular unpublished data on age distributions.

**Results:**

We identified 128 articles containing 227 country datasets (61 age distributions, 71 incidence rates and 95 CFRs). The median age of intussusception ranged from 29 weeks in Africa (83% of cases in the first year of life) to 70 weeks in the Western Pacific region (35% of cases in the first year of life). The median (range) annual incidence of intussusception hospital admissions per 100 000 aged <1 year ranged from 34 (13–56) in Africa to 90 (9–380) in the Western Pacific region. We found extreme differences between the CFRs in Africa (1 death in every 10 hospital admissions) and the rest of the world (fewer than 1 death in every 100–2000 hospital admissions).

**Conclusion:**

Intussusception epidemiology varies by country and region. Understanding and recognizing these differences will be important when assessing the potential number of intussusception cases associated with rotavirus vaccines.

**Key Messages**

- Intussusception incidence varies substantially by country and region, highlighting the need to assess the benefits and risks of rotavirus vaccines at the national, rather than global, level.
- Administering the first dose of rotavirus vaccination at birth, rather than at 6 weeks, could avoid nearly all vaccine-related cases of intussusception.
- We found extreme differences between case-fatality ratios in Africa (1 death in every 10 hospital admissions) and the rest of the world (fewer than 1 death in every 100–2000 hospital admissions), highlighting the urgent need for strategies to reduce the time between onset of symptoms and presentation at hospital.

## Introduction

Intussusception is the main cause of bowel obstruction in children aged <5 years. It occurs when a segment of the intestine telescopes or folds back on itself.^1^ This blocks the passage of food and liquid through the intestine and restricts the supply of blood to the affected area. Some cases of intussusception will spontaneously resolve without treatment, but delayed diagnosis can lead to perforation and infection in the lining of the abdominal cavity (peritonitis). Peritonitis can cause severe abdominal pain, fever, shock and death.^2^ In high-income countries, most children are diagnosed quickly with ultrasound or radiograph and the bowel will return to normal after injecting a liquid or gas into the rectum (enema). In more severe cases, surgery is usually very successful. However, in parts of Africa and elsewhere, many children will die before reaching healthcare; for those who do reach healthcare, surgery is often the primary method of diagnosis and treatment, leading to death in ∼10% of cases.^3^

The cause of intussusception is usually unclear, but infections that cause swelling in the bowel wall may be associated.^4^ In some countries that have introduced oral rotavirus vaccines, a small but elevated risk of intussusception has been reported in the first few weeks after administration of the first and second doses. To limit the scale of potential vaccine-related intussusception cases, the vaccine manufacturers have recommended administration of the first dose before ∼15 weeks of age (and the final dose before ∼32 weeks of age) when the background rate of intussusception is relatively low.^5^

Published intussusception incidence rates, age distributions and case-fatality ratios (CFRs) have been reviewed and published by the World Health Organization (WHO) (1960–2002)^2^ and Jiang *et al.* (2002–12)^3^ but there are a number of reasons why this evidence should now be updated. First, many new studies have been published since 2012. Second, age distributions and incidence rates were previously restricted to children aged <1 year despite many cases being observed between the ages of 1.0 and 5.0 years in some settings.^6–8^ Third, CFRs were estimated for a variety of age groups rather than a standard (e.g. <5 years), making it difficult to compare countries and estimate ratios in countries with no data. Fourth, published intussusception age distributions are rarely published by week of age. Obtaining data from authors/investigators at this level of age granularity will provide inputs that are critical for modelling the number of excess intussusception cases that could be associated with different rotavirus vaccination schedules in different settings. This calculation involves combining estimates of the background number of intussusception cases in each week of age with data on the coverage of each dose in each week of age^9^ and the relative risk of intussusception in the first, second and third weeks after each dose is administered.^10^

In this paper, we provide an updated global review of the incidence, age distribution and case fatality of intussusception hospital admissions among children aged <5 years of age, before the introduction of rotavirus vaccination.

## Methods

### Search strategy

We sought information from published research articles on intussusception incidence rates, age distributions and CFRs in children aged <5 years, before the introduction of rotavirus vaccination. We included all studies published between January 2002 and June 2012 that were identified in a previous review by Jiang *et al.*^3^ We then added all relevant studies published between June 2012 and January 2018, identified from a new global systematic literature review. This review used search terms that were consistent with the review by Jiang *et al.*, i.e. ‘intussusception’ or ‘intestinal invagination’. It was conducted in accordance with PRISMA guidelines. We searched PubMed, EMBASE, MEDLINE and Cochrane Library. We restricted the search to articles published in English, French, Spanish and Polish. To increase the relevance of our analysis, we excluded all studies published before January 2002. We therefore excluded all studies from an earlier review by WHO (1960–2002).^2^ All titles and abstracts identified by the systematic review were screened for inclusion by two reviewers (M.H.A. and L.K.S) using Distiller software. Any disagreements were resolved by a third reviewer (A.D.C.). Articles were excluded if: (i) it was not possible to extract data for the years prior to rotavirus-vaccine introduction; (ii) the data period ended prior to the year 2000; (iii) cases were not coded as ICD9-560, ICD10-K56.1, Brighton Collaboration Level 1 (BCL1) or defined clinically as intussusception; (iv) there were fewer than 35 cases (for age-distribution fitting); (v) more recent data were published elsewhere for the same population/location (for incidence rates); (vi) they described animal studies; (vii) they described individual case reports; (viii) they focused on a specific subgroup of cases, e.g. only patients with chronic or recurrent intussusception; (ix) they were conducted in special populations, e.g. HIV-positive; or (x) the study had a high risk of bias. All remaining studies were assigned very low, low or medium risk of bias (see Supplementary AppendixTable A1, available as Supplementary data at *IJE* online). If information from multiple countries was published as a single data point, it was included if all countries were from the same WHO region and it was clear that none of the country-level data were reported elsewhere.

### Data extraction

We compiled a database containing information on intussusception incidence rates, age distributions and CFRs in children aged <5 years. For all studies, we extracted the country, subnational location, study design, case definition, period of data collection and age range. To obtain more granular age distributions, we emailed an invitation letter to all authors who were listed in the Jiang *et al.* review (2002–12)^3^ and all authors identified in the new systematic review (2012–18). We invited each author to share a spreadsheet table with counts of intussusception hospital admissions by week of age up to 5.0 years. If the authors did not respond, then we extracted the age distributions published in the research article. We also extracted the published incidence rate and the number of intussusception cases and deaths.

A country dataset was defined as any dataset with hospitalized patients before the introduction of rotavirus vaccine, taken from a single study in a single country and reporting on a single outcome, e.g. age distribution, incidence rate and CFR. If a study included multiple years and multiple sites, then all pre-vaccination years and subnational sites were aggregated and included in the same country dataset. The main outcome/presentation was hospital admissions, but we also included emergency room visits if admissions were not reported in the same study.

### Age distribution of intussusception hospital admissions <5 years

Age distributions were fitted to all studies that had at least three age bands below the age of 1.0 year to ensure there was enough information to inform the shape of the age distribution. Age distributions that did not capture the entire age range <5 years (e.g. <1 year, <2 years) were adjusted to ensure that each country dataset had a realistic right-hand tail prior to fitting. To do this, we calculated the median cumulative proportion of intussusception cases that were reported to have occurred by ages 1, 2, 3, 4 and 5 years in each WHO region, using a subset of country datasets that had a full set of counts in each single year of age up to 5.0 years. The median proportions in each WHO region were then used to estimate the expected number of intussusception cases in each missing single year of age <5 years.

We fitted a range of parametric age distributions <5 years (Gamma, Weibull, Lognormal, Log Logistic, Burr) to each country dataset using Non-linear Least Squares (NLS) and Maximum Likelihood Estimation (MLE). Each distribution was compared using the Root Mean Squared Error (RMSE), Mean Absolute Error (MAE), goodness-of-fit statistics (Kolmogorov-Smirnov, Cramer-von Mises, Anderson-Darling) and goodness-of-fit criteria (Akaike’s Information Criterion, Bayesian Information Criterion).

We estimated the median age, interquartile age range and cumulative proportion of intussusception cases estimated to have occurred by standard ages between birth and age 5.0 years. All analyses were conducted using R (R version 3.4.1, R Foundation for Statistical Computing, Vienna, Austria) and the R packages *MASS*, *nloptr*, *fitdistrplus*, *actuar* and *mutil*.

Results were stratified by the following WHO regions: the Americas (AMR); Africa (AFR); Eastern Mediterranean (EMR); Europe (EUR); Southeast Asia (SEA); and Western Pacific (WPR).^11^

### Incidence of intussusception hospital admissions <5 years

For country datasets that did not capture the entire age range <5 years (e.g. <1 year, <2 years) we calculated an adjusted incidence rate per 100 000 per year <5 years. First, we estimated the expected number of cases in each missing single year of age <5 years using the median cumulative proportion of intussusception cases by ages 1, 2, 3, 4 and 5 years in each WHO region. These median proportions were based on the entire set of fitted parametric age distributions <5 years, so included more studies than those used to adjust the right-hand tails of age distributions prior to fitting (see above). Second, we inflated the denominator based on the ratio between the size of the population in the reported age group and the under-five age group. Ratios were determined using UNPOP population data for ages 0, 1, 2, 3 and 4 years in the period 2010–15.^12^ Results were stratified by WHO region.

### In-hospital intussusception CFRs <5 years

We used three alternative approaches to estimate CFRs for each country dataset and each WHO region. First, we calculated age-unadjusted CFRs by dividing reported deaths by reported cases in each country and WHO region. Second, we calculated age-unadjusted CFRs and 95% confidence intervals (CIs) for each country and WHO region using meta-analysis. For meta-analysis, we used the *metaprop_one* and *metareg* commands in Stata version 15.1 (StataCorp. 2017. Stata Statistical Software: Release 15. College Station, TX: StataCorp LLC). With *metaprop_one*, we chose the logit option, so that binomial distributions were used to model within-study variability. Logistic models with random intercepts were fitted and the variability of the random intercepts indicated heterogeneity. We also ran a separate meta-analysis with results pooled by the national under-five mortality quintile. Quintiles were based on the 2010–15 under-five mortality rates published by the UNPOP (2017 Revision).^12^ Third, we calculated age-adjusted CFRs by converting CFRs that did not extend the full 5-year age range (e.g. <1 year, <2 years) into a CFR aged <5 years. To do this, we first calculated the expected number of intussusception cases in the missing age range (e.g. 12–59 months) using the median cumulative proportion of cases expected to occur by each age in each WHO region. These estimates were based on the full set of fitted parametric age distributions <5 years in each WHO region. To estimate CFRs in the missing age range, the ratio of difference between the CFR in the known and missing age range was assumed to be the same as the ratio of difference between the probability of dying from any cause in the known and missing age ranges during a single year of life. These probabilities were derived from country-specific life tables for the period 2010–15.^12^

## Results

### Search results

**Figure 1.**
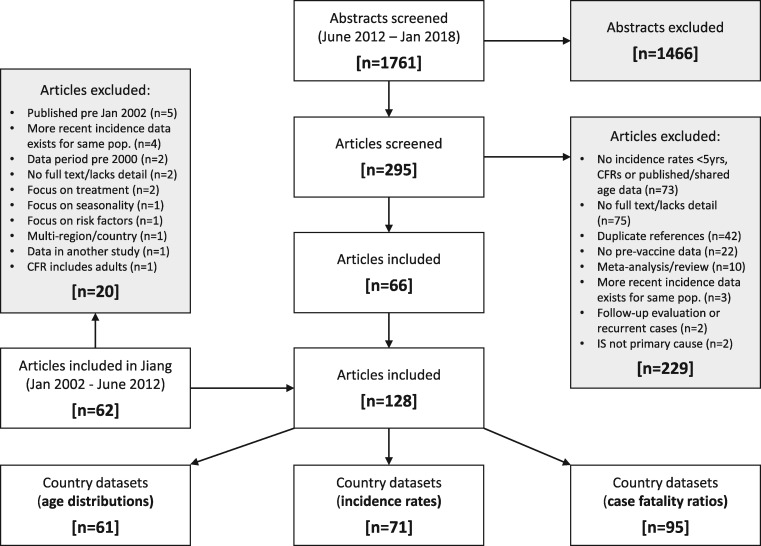
Flow diagram to show search for country datasets.

After exclusions, we identified 128 articles (Figure 1). We included 62 articles from the Jiang *et al.* review (2002–12) and 66 articles from the new search (2012–18). There were 61 country datasets with age distributions, 71 with incidence rates and 95 with CFRs (Supplementary AppendixTable A1, available as Supplementary data at *IJE* online). We obtained additional age granularity from authors in around half (28/61) of the country datasets with age distributions. We included post-vaccination age distribution and CFR data for one study in Africa^13^ because it included high-quality prospective data for several African countries and found no elevated risk of intussusception. We also included data from China and Vietnam despite uncertainties about the level of vaccine use in the private market. We included data from four studies that were published after the end date of the systematic review.^13–16^

### Age distribution of intussusception hospital admissions <5 years

**Figure 2.**
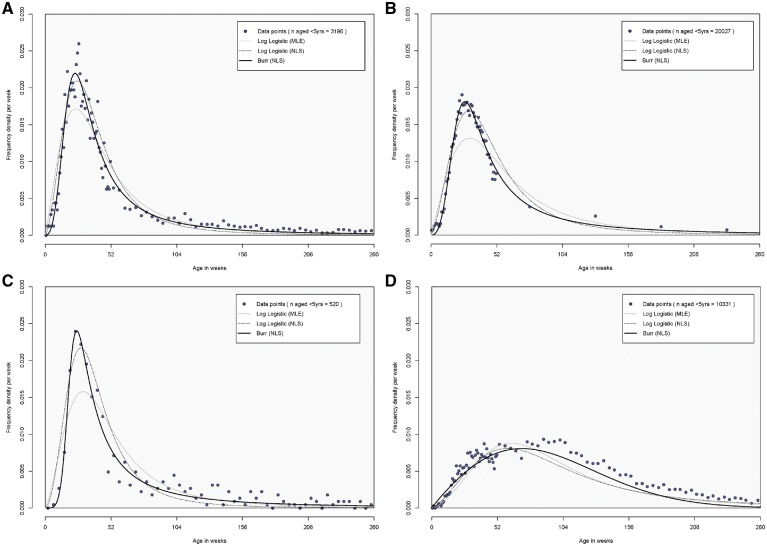
Comparison of fitted age distributions for intussusception hospital admissions among children aged <5 years for selected country datasets. (**A**) England (2002–12); (**B**) USA (1994–2004); (**C**) Hong Kong (1997–2011); (**D**) Taiwan (1998–2013).

Prior to fitting, we used a subset of 31 datasets with complete counts in each single year of life between birth and age 5.0 years (Supplementary Appendix Table A2, available as Supplementary data at *IJE* online) to estimate realistic right-hand tails in the datasets with incomplete age distributions <5 years. Using MLE, in most of the 61 country datasets, the *Burr* distribution had the most favourable goodness-of-fit statistics and goodness-of-fit criteria compared with the *Weibull*, *Lognormal*, *Gamma* and *Log Logistic* distributions. However, the fits based on NLS had a lower overall RMSE and a much better visual fit to the data than MLE, particularly around the peak of the age distribution (Figure 2). The *Burr* distribution had more favourable RMSE and MAE statistics than the *Log Logistic* distribution in over 80% of the country datasets and a better visual fit to distributions with long tails. Our preference was to use a standard approach to fitting, summarizing and extrapolating curves to countries without data so we fitted the *Burr* distribution to all 61 datasets^17^^,^^18^ (Supplementary Appendix Table A3, available as Supplementary data at *IJE* online).

In the African, Eastern Mediterranean and Southeast Asian regions, ≥80% of intussusception hospital admissions were in the first year of life, compared with 63% in the Americas, 54% in Europe and 35% in the Western Pacific region. The median proportion of cases occurring by 15 weeks of age ranged from 2.4% in the Western Pacific region to 6.8% in the Eastern Mediterranean region (Table 1). There was substantial within-region variation in the European and the Western Pacific regions (Supplementary Appendix Table A4, available as Supplementary data at *IJE* online).

**Table 1.** Incidence rates, age distributions and CFRs among intussusception hospital admissions aged <5 years in different WHO regions before the introduction of rotavirus vaccination

	Africa	Americas	Eastern Mediterranean	Europe	Southeast Asia	Western Pacific
Annual incidence (range) per 100 000 per year						
Number of country datasets	2	20	3	19	7	20
<1 year	34 (13–56)	36 (4–105)	78 (51–95)	41 (12–138)	77 (18–253)	90 (9–380)
<5 years	8 (3–14)	11 (1–34)	19 (13–23)	14 (4–49)	19 (4–61)	52 (5–196)
Median age (inter-quartile range) of admission						
Number of country datasets	14	7	4	13	7	16
Age in weeks	29 (22–43)	41 (27–69)	30 (22–42)	47 (29–89)	33 (24–47)	70 (42–126)
Median parameters of the Burr distribution						
Shape 1 (γ)	4.80	3.62	4.08	2.81	4.17	2.65
Shape 2 (α)	0.44	0.39	0.65	0.44	0.55	0.51
Scale (θ)	22.20	26.15	25.46	29.33	26.39	46.83
Median cumulative % of admissions by age^a^						
6 weeks	0.1%	0.2%	0.2%	0.5%	0.1%	0.2%
10 weeks	0.9%	1.2%	1.4%	2.0%	1.0%	0.8%
14 weeks	4.4%	3.8%	5.3%	5.0%	3.7%	2.0%
15 weeks	6.0%	4.8%	6.8%	6.0%	4.9%	2.4%
1 month	0.0%	0.1%	0.0%	0.2%	0.0%	0.1%
2 months	0.5%	0.7%	0.8%	1.4%	0.5%	0.6%
3 months	3.2%	3.0%	4.0%	4.1%	2.8%	1.7%
4 months	11.0%	7.7%	11.5%	8.6%	8.5%	3.5%
5 months	24.3%	14.8%	23.7%	14.4%	18.3%	6.1%
6 months	39.4%	23.5%	37.9%	21.0%	30.7%	9.3%
7 months	52.5%	32.3%	51.4%	27.7%	43.3%	13.1%
8 months	62.7%	40.5%	62.4%	34.2%	54.4%	17.4%
9 months	70.2%	47.7%	70.8%	40.1%	63.3%	21.8%
10 months	75.9%	53.9%	77.1%	45.5%	70.2%	26.3%
11 months	80.1%	59.0%	81.8%	50.2%	75.6%	30.8%
12 months	83.4%	63.4%	85.4%	54.4%	79.8%	35.1%
18 months	92.9%	78.9%	94.8%	70.8%	91.9%	55.6%
24 months	96.1%	85.9%	97.6%	79.2%	95.8%	68.1%
36 months	98.3%	92.0%	99.2%	87.3%	98.4%	80.9%
48 months	99.1%	94.7%	99.6%	91.0%	99.2%	87.0%
60 months	99.4%	96.1%	99.8%	93.2%	99.5%	90.3%
Case-fatality ratio						
Number of country datasets	27	13	4	17	16	18
Pooled number of deaths	407	117	3	18	8	6
Pooled number of cases	3, 739	47, 616	368	10, 365	2, 467	11, 606
CFR (unadjusted)	10.89%	0.25%	0.82%	0.17%	0.32%	0.05%
CFR (adjusted to age <5 years)	10.08%	0.17%	0.81%	0.17%	0.32%	0.03%
CFR (age-unadjusted) meta-analysis	11.50%	0.41%	0.46%	0.20%	0.27%	0.05%
CFR (age-unadjusted) meta-analysis, 95% CI	(7.24–17.78%)	(0.11–1.54%)	(0.02–8.74%)	(0.05–0.89%)	(0.03–2.48%)	(0.02–0.12%)

a The best-fitting parameters for each WHO region were calculated by refitting Burr distributions to the pooled proportion of intussusception admissions in each week of age <5 years. Cumulative proportions below 100% by age 60 months indicate that some cases are estimated to occur after this age. The Burr distribution (Burr type XII) has shape 1 (γ), shape 2 (α) and scale (θ), all of which must be positive values (note: the Burr distribution becomes the Log Logistic distribution when the shape 2 parameter equals 1.0). The cumulative distribution function (cdf) of the Burr distribution is:

$f\left(x\right)=1-{\left[1+{\left(\frac{x}{\theta }\right)}^{\gamma }\right]}^{-\alpha }.$

The three parameters (γ, α, θ) can also be used to derive the probability density function, median, mode, mean and variance. These equations are described in detail elsewhere.^18^

**Figure 3:**
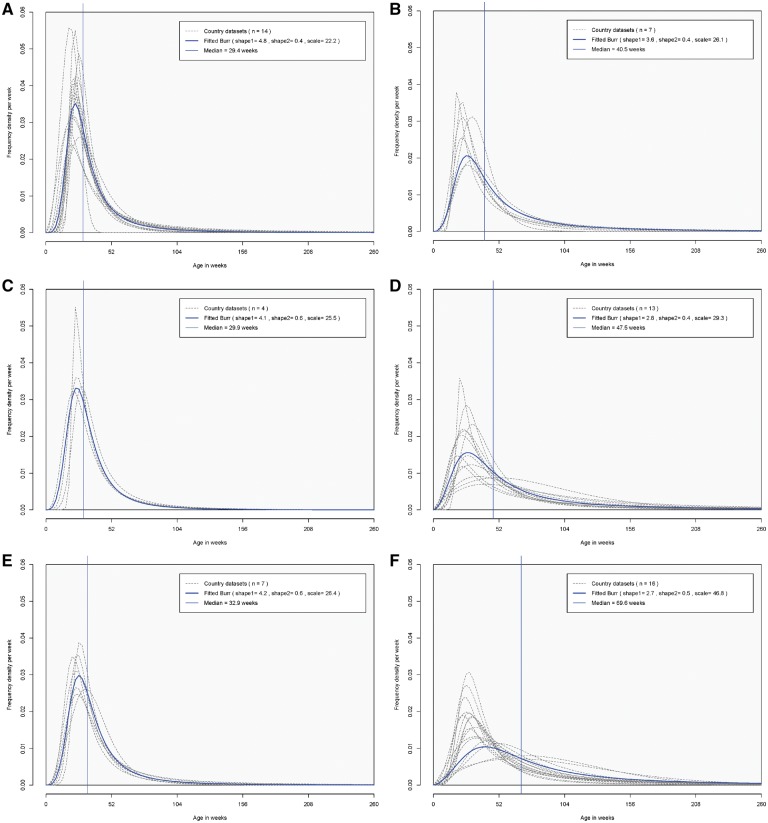
Age distribution of intussusception hospital admissions among children aged <5 years by WHO region. (**A**) Africa; (**B**) Americas; (**C**) Eastern Mediterranean; (**D**) Europe; (**E**) Southeast Asia; (**F**) Western Pacific.

Median (IQR) ages of intussusception hospital admissions were: 29 (22–43) weeks in Africa, 30 (22–42) weeks in the Eastern Mediterranean, 33 (24–47) weeks in Southeast Asia, 41 (27–69) weeks in the Americas, 47 (29–89) weeks in Europe and 70 (42–126) weeks in the Western Pacific region (Figure 3).

### Incidence of intussusception hospital admissions <5 years

**Figure 4.**
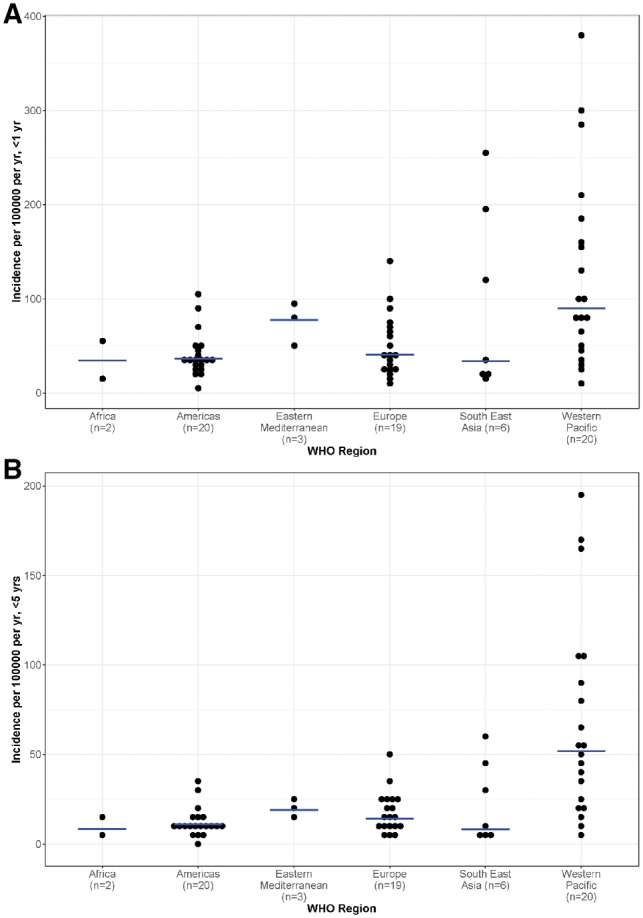
Incidence of intussusception hospital admissions by WHO region. (**A**) <1 year; (**B**) <5 years.

The median (range) annual incidence rate of intussusception hospital admissions was 8 (3–14) per 100 000 aged <5 years in Africa, 11 (1–34) in the Americas, 19 (13–23) in the Eastern Mediterranean region, 14 (4–49) in Europe, 19 (4–61) in Southeast Asia and 52 (5–196) in the Western Pacific region (Figure 4 and Table 1). Incidence rates in the Western Pacific region ranged from ∼5 in Malaysia to ∼200 in Nha Trang, Vietnam. Incidence rates above 70 per 100 000 per year <5 years were found in Japan, South Korea and Vietnam (Supplementary Appendix Table A5, available as Supplementary data at *IJE* online). Globally, we found no correlation (*R*^2^ < 0.1%) between the adjusted incidence rates <5 years and the under-five mortality rate for the period 2010–15. Median incidence rates among children aged <1 year were above 75 per 100 000 per year in Southeast Asia and in the Eastern Mediterranean and Western Pacific regions (Table 1 and Figure 4).

### In-hospital intussusception CFRs <5 years

Pooled CFRs in each WHO region varied according to the method used (Table 1). The CFR (95% CI for each region) based on age-unadjusted meta-analysis was 11.5% (7.24–17.78%) in Africa, 0.41% (0.11–1.54%) in the Americas, 0.46% (0.02–8.74%) in the Eastern Mediterranean region, 0.20% (0.05–0.89%) in Europe, 0.27% (0.03–2.48%) in Southeast Asia and 0.05% (0.02–0.12%) in the Western Pacific region, but there was variation within each region (Table 1, Supplementary Appendix Table A6, available as Supplementary data at *IJE* online). We chose to stratify by WHO region rather than under-five mortality quintile. Both had similar explanatory power in meta-regression (*p* < 0.005) but WHO region was consistent with the stratification used for age distributions and incidence rates, and had more favourable between-group heterogeneity (*p* = 0.001 vs *p* = 0.003). The strongest predictor of CFRs was whether a country was based in the region of Africa or not. Stratifying by study age group gave no evidence of heterogeneity (*p* = 0.7732) so CFRs were not adjusted for age in meta-analysis.

## Discussion

This analysis provides an important update to the existing global evidence on intussusception incidence rates, age distributions and CFRs prior to rotavirus-vaccine introduction. More than half of the research articles (67/129) included in our analysis were published after the previous review by Jiang *et al*.^3^ In addition, we have provided incidence rates and CFRs that are both unadjusted and adjusted to a standard age group (<5 years), allowing the totality of evidence to be included and compared across countries. We have also benefited from the generosity of many study investigators, who were able to share a more precise breakdown of their age data. We fitted standard parametric curves to all datasets using statistically robust methods that will allow estimates of intussusception cases in each week of age <5 years.

Our analysis found that the median annual incidence of intussusception ranges from 34 (African region) to 90 (Western Pacific region) per 100 000 children aged <1 year. The previous review by Jiang *et al.* estimated a global incidence of 74 in the same age range.^3^ Several Western Pacific countries reported very high incidence rates. The reason for this is unclear. A case–control study in Vietnam and Australia found no association between intussusception and diet or living conditions but did find a strong association with adenovirus, suggesting this may play a role in the aetiology of intussusception.^19^ High incidence may also be associated with a high rate of recurrent cases. However, recurrent cases represented <15% of total intussusception hospital admissions in each of the 32 studies where this proportion was reported. In one study from the Western Pacific region, we were able to exclude the recurrent cases and the median age was still high (67 weeks).^16^ There were very few incidence-rate data points from the African region (two data points) and Eastern Mediterranean region (three data points). Relatively low incidence rates from these regions may simply reflect the fact that many children did not reach hospital. For all datasets, we accepted the definition of intussusception provided by the authors. However, different definitions and coding systems could have led to important differences in results. One study in Bangladesh found a very large range of possible incidence rates (0–97 per 100 000 per year, <2 years) depending on whether the study was retrospective or prospective, and whether cases were probable or confirmed.^20^ Around one-third (25/71) of the country datasets with incidence rates were based on studies with a prospective design and most (63/71) used specific ICD codes or BCL case definitions. We excluded incidence rates if a more recent data point was reported in the same population/location but there is some evidence that incidence rates may be decreasing over time. In England, estimates of the annual incidence of intussusception hospital admissions <1 year were 66 for the period 1993–95,^21^ 30 for the period 2002–12 (unpublished from ^10^) and 24 for the period 2008–09.^22^ In California, the rate declined from in 51 in 1985–97 to 37 in 2000–05.^23^

Countries with higher incidence rates tended to have a higher median age. This effect was largely driven by countries in the Western Pacific region where high incidence rates and high median ages were commonly reported. Our analysis of age distributions found median ages ranging from 29 weeks in Africa (83% of cases in the first year of life) to 70 weeks in the Western Pacific region (35% of cases in the first year of life). However, regional medians may mask important within-region variation and, in some regions, the data are from a relatively limited subset of countries that may not be representative of all countries within the region. For example, the country datasets in Switzerland and Germany had a much higher median age of intussusception than the median for the European region and many of the studies from Africa were from Anglophone rather than Francophone countries. We did not fit granular age distributions to datasets with fewer than 35 cases to ensure the fits were reliable, but this meant excluding some studies from e.g. Malawi and Rwanda. In a small number of studies with very high median ages, there was some evidence of a second peak shortly after the first.^6^^,^^24–27^ In these studies, our best-fitting age distributions under-estimated cases among older children (Figure 2) but the fits were generally very good in the age range of importance to measuring vaccine-associated intussusception events. The observed decreases in incidence <1 year in England and the USA partly reflect a shift in intussusception cases to older age groups over time. National Inpatient Survey (NIS) data representing 20% of hospitals in the USA have shown that the proportion of under-five intussusception admissions aged <1 year has declined from 62% in 1994 to 50% in 2004 and continues to decline in the post-vaccine era (unpublished from ^28^).

We found extreme differences between the CFRs in Africa (1 death in every 10 hospital admissions) and the rest of the world (fewer than 1 death in every 100–2000 hospital admissions). In Africa, many children arrive late to hospital in advanced stages of illness. Compared with other parts of the world, a very high proportion of cases are diagnosed and treated with surgery.^29^^,^^30^ Strategies are urgently needed to reduce the time between onset of symptoms and presentation at hospital. Removing barriers to timely access in Africa would dramatically reduce the risk of complications and other contraindications that prohibit the use of lower-risk treatment options, such as enemas. Investment is also needed to ensure hospitals have the appropriate imaging equipment (ultrasound, radiograph) and staff required to implement lower-risk treatment.^18^ We did not formally evaluate the proportion of cases receiving different types of diagnostics and treatment in different settings. This would be a worthwhile follow-up analysis and would inform estimates of the costs of intussusception treatment in different settings.

Our analysis excludes children without access to hospital. Most children with intussusception will die if left untreated, but an uncertain proportion will spontaneously resolve without treatment.^31–35^ Better estimates of access to hospital, and spontaneous recovery without treatment, would be needed to generate realistic estimates of the CFR among all cases of intussusception.^36^ These adjustments could be influential in Africa, leading to higher overall CFRs than reported in our analysis of hospital admissions.

In the context of rotavirus vaccines, a recent study in Africa encouragingly found no intussusception risk associated with a live oral rotavirus vaccine,^13^ but an elevated risk has been found with the same vaccine, and other oral rotavirus vaccines, in other parts of the world. The authors provide several reasons why the risk may have been lower in the African study including less frequent shedding of the vaccine strain in lower-income settings, co-administration with oral polio vaccination and administering the vaccine at a younger age (6 and 10 weeks).^37^ Our analysis provides inputs that are critical to assess the number of excess intussusception cases that could be associated with different rotavirus vaccination schedules in different settings. It is important to evaluate this at the national level given the substantial country-level variation observed in age distributions, incidence rates and CFRs, as well as vaccine schedules, coverage and timeliness.^5^^,^^9^ Avoiding the peak age of intussusception is important for the design of rotavirus vaccination schedules because the absolute risk is relative to the background incidence. Our analysis encouragingly found that less than 5% of hospital admissions occurred before age 15 weeks (median of all 71 datasets) when the first dose is typically administered. If the first dose of rotavirus vaccination could be administered at birth,^38^ this would avoid nearly all of the background incidence. Large-scale post-licensure studies are needed to assess whether this strategy can substantially lower the risk of intussusception, without reducing the benefits of rotavirus vaccination. We excluded case counts recorded in the first week of life. Several datasets reported a suspiciously high number in this age group, which may be related to errors in the recording of the date of birth/admission or errors in the diagnosis. For example, necrotizing enterocolitis and other neonatal congenital problems may be misdiagnosed as intussusception.^39^

## Conclusion

The incidence, age distribution and case fatality of intussusception hospital admissions vary by region. Understanding and recognizing these differences will be important when assessing the number of intussusception cases that could be associated with different rotavirus vaccination schedules in different settings.

## Funding

This work was supported by the Bill and Melinda Gates Foundation (BMGF) (OPP1147721). BMGF had no role in the writing of the manuscript or the decision to submit it for publication.

## Supplementary Material

dyz028_Supplementary_Data
