# Supplementary material for: Update on the global epidemiology of intussusception: a systematic review of incidence rates, age distributions and case-fatality ratios among children aged <5 years, before the introduction of rotavirus vaccination
Source: Int J Epidemiol. 2019 Mar 16;48(4):1316–26. doi: 10.1093/ije/dyz028 (PMC6693807; doi:10.1093/ije/dyz028)
Supplement: dyz028_Supplementary_Data [file dyz028_supplementary_data.docx]

**Appendix to:**

*Update on the global epidemiology of intussusception: a systematic review of incidence rates, age distributions and case fatality ratios among children aged <5 years, before the introduction of rotavirus vaccination*

Clark A, Hasso-Agopsowicz M, Kraus M, Stockdale L, Sanderson CFB, Parashar U, Tate J.

**Appendix Table 1. Characteristics of country datasets included in the analysis**

| **Region** | **Country** | **Location** | **From** | **To** | **Prosp. study design?** | **Specific ICD code or BCL?** | **Included in Jiang *et al* 2012?** | **Author shared granular age data?** | **Single year age counts <5yrs?** (Appendix Table 2) | **Fitted age distro. <5yrs?**  (Appendix Table 3/4) | **Age-specific**  **incidence rate?**  (Appendix Table 5) | **CFR?**  (Appendix Table 6) | **Risk of bias*** | **First Author** | **Year** | **Ref** |
| --- | --- | --- | --- | --- | --- | --- | --- | --- | --- | --- | --- | --- | --- | --- | --- | --- |
|  |  |  |  |  |  |  |  |  |  |  |  |  |  |  |  |  |
| TOTAL |  |  |  |  | 42 | 100 | 63 | 33 | 31 | 61 | 71 | 95 |  |  |  |  |
|  |  |  |  |  |  |  |  |  |  |  |  |  |  |  |  |  |
| AFR |  |  |  |  | 11 | 8 | 10 | 9 | 5 | 14 | 2 | 27 |  |  |  |  |
| AMR |  |  |  |  | 12 | 27 | 10 | 6 | 3 | 7 | 20 | 13 |  |  |  |  |
| EMR |  |  |  |  | 1 | 3 | 4 | 2 | 3 | 4 | 3 | 4 |  |  |  |  |
| EUR |  |  |  |  | 8 | 25 | 17 | 6 | 5 | 13 | 19 | 17 |  |  |  |  |
| SEA |  |  |  |  | 6 | 14 | 8 | 1 | 6 | 7 | 7 | 16 |  |  |  |  |
| WPR |  |  |  |  | 4 | 23 | 14 | 9 | 9 | 16 | 20 | 18 |  |  |  |  |
|  |  |  |  |  |  |  |  |  |  |  |  |  |  |  |  |  |
| AFR | Africa | 10 countries | 1993 | 2003 |  |  | Yes |  |  | Yes |  | Yes | Medium | Steele | 2012 | (1) |
| AFR | Ethiopia | Addis Ababa | 2011 | 2014 |  |  |  |  |  | Yes |  | Yes | Low | Gadisa | 2016 | (2) |
| AFR | Ethiopia | 6 hospitals | 2013 | 2016 | Yes | Yes |  | Yes |  | Yes |  | Yes | Medium | Tate | 2018 | (3) |
| AFR | Ghana | Kumasi | 2004 | 2007 | Yes |  | Yes |  |  |  |  | Yes | Low | Abantanga | 2008 | (4) |
| AFR | Ghana | Accra | 2008 | 2009 |  |  | Yes |  | Yes | Yes |  |  | Low | Enweronu-Laryea | 2012 | (5) |
| AFR | Ghana | 2 hospitals | 2012 | 2016 | Yes | Yes |  | Yes |  | Yes |  | Yes | Medium | Tate | 2018 | (3) |
| AFR | Kenya | Eldoret | 2000 | 2003 |  |  | Yes |  |  |  |  | Yes | Medium | Kuremu | 2004 | (6) |
| AFR | Kenya | National | 2002 | 2013 |  | Yes |  | Yes | Yes | Yes |  | Yes | Low | Omore | 2016 | (7) |
| AFR | Kenya | Bomet | 2009 | 2013 |  |  |  |  |  |  |  | Yes | Low | Ooko | 2016 | (8) |
| AFR | Kenya | 5 hospitals | 2014 | 2016 | Yes | Yes |  | Yes | Yes | Yes |  | Yes | Medium | Tate | 2018 | (3) |
| AFR | Malawi | 4 hospitals | 2013 | 2016 | Yes | Yes |  | Yes |  |  |  | Yes | Medium | Tate | 2018 | (3) |
| AFR | Nigeria | Lagos | 1995 | 2001 | Yes |  | Yes |  | Yes | Yes |  | Yes | Medium | Bode | 2008 | (9) |
| AFR | Nigeria | Enugu | 2008 | 2009 |  |  | Yes |  |  |  |  | Yes | Low | Ekenze | 2010 | (10) |
| AFR | Nigeria | Enugu | 1998 | 2007 |  |  | Yes |  |  |  |  | Yes | Low | Ekenze | 2011 | (11) |
| AFR | Nigeria | Enugu | 2009 | 2013 |  |  |  |  |  |  |  | Yes | Low | Ekenze | 2015 | (12) |
| AFR | Nigeria | Ibadan | 2002 | 2011 |  |  |  |  |  | Yes |  | Yes | Low | Ogundoyin | 2016 | (13) |
| AFR | Nigeria | Ile-Ife | 1993 | 2011 |  |  |  |  |  | Yes |  | Yes | Low | Talabi | 2013 | (14) |
| AFR | Rwanda | Kigali | 2009 | 2012 |  |  |  |  |  |  |  | Yes | Low | Ngendahayo | 2014 | (15) |
| AFR | South Africa | Johannesburg | 2007 | 2010 | Yes |  |  | Yes |  |  |  | Yes | Low | Carapinha | 2016 | (16) |
| AFR | South Africa | 9 hospitals | 1998 | 2003 |  |  | Yes |  |  |  | Yes | Yes | Low | Moore | 2010 | (17) |
| AFR | South Africa | Bloemfontein | 2003 | 2011 |  |  |  |  |  |  |  | Yes | Low | Venter | 2013 | (18) |
| AFR | South Africa | Not reported | 1996 | 2001 |  |  | Yes |  |  |  |  | Yes | Low | Wiersma | 2004 | (19) |
| AFR | Tanzania | Dar es Salaam | 2000 | 2004 |  |  | Yes |  |  |  |  | Yes | Low | Carneiro | 2004 | (20) |
| AFR | Tanzania | Mwanza | 2010 | 2012 | Yes |  |  |  | Yes |  |  | Yes | Low | Chalya | 2014 | (21) |
| AFR | Tanzania | 7 hospitals | 2013 | 2016 | Yes | Yes |  | Yes |  | Yes |  | Yes | Medium | Tate | 2018 | (3) |
| AFR | Zambia | 9 hospitals | 2007 | 2011 |  |  |  |  |  | Yes | Yes | Yes | Low | Mpabalwani | 2014 | (22) |
| AFR | Zambia | 4 hospitals | 2013 | 2016 | Yes | Yes |  | Yes |  | Yes |  | Yes | Medium | Tate | 2018 | (3) |
| AFR | Zimbabwe | Harare | 2014 | 2016 | Yes | Yes |  | Yes |  | Yes |  | Yes | Medium | Tate | 2018 | (3) |
| AMR | Argentina | Mendoza | 2003 | 2005 | Yes | Yes |  |  |  |  | Yes |  | V. Low | Sáez-Llorens | 2013 | (23) |
| AMR | Brazil | Not reported | 2003 | 2005 | Yes | Yes |  |  |  |  | Yes |  | V. Low | Sáez-Llorens | 2013 | (23) |
| AMR | Brazil | National | 2001 | 2006 |  | Yes |  | Yes | Yes | Yes |  |  | Medium | Teles | 2015 | (24) |
| AMR | Canada | Toronto | 2002 | 2006 |  |  |  |  |  |  |  | Yes | Low | Bailey | 2007 | (25) |
| AMR | Canada | Ontario | 2010 | 2010 |  |  |  |  |  |  | Yes |  | Low | Ducharme | 2013 | (26) |
| AMR | Chile | Santiago | 2000 | 2001 |  | Yes | Yes |  |  | Yes | Yes | Yes | V. Low | O’Ryan | 2003 | (27) |
| AMR | Chile | Not reported | 2003 | 2005 | Yes | Yes |  |  |  |  | Yes |  | V. Low | Sáez-Llorens | 2013 | (23) |
| AMR | Colombia | Cali | 2003 | 2005 | Yes | Yes |  |  |  |  | Yes |  | V. Low | Sáez-Llorens | 2013 | (23) |
| AMR | Costa Rica | San Jose | 2003 | 2005 | Yes | Yes |  |  |  |  | Yes |  | V. Low | Sáez-Llorens | 2013 | (23) |
| AMR | Dominican Rep. | Santo Domingo | 2003 | 2005 | Yes | Yes |  |  |  |  | Yes |  | V. Low | Sáez-Llorens | 2013 | (23) |
| AMR | Honduras | Tegucigalpa | 2003 | 2005 | Yes | Yes |  |  |  |  | Yes |  | V. Low | Sáez-Llorens | 2013 | (23) |
| AMR | Latin America | 11 countries | 2003 | 2005 | Yes | Yes |  |  |  | Yes |  | Yes | V. Low | Sáez-Llorens | 2013 | (23) |
| AMR | Mexico | Mexico City | 2003 | 2005 | Yes | Yes |  |  |  |  | Yes |  | V. Low | Sáez-Llorens | 2013 | (23) |
| AMR | Nicaragua | Managua | 2003 | 2005 | Yes | Yes |  |  |  |  | Yes |  | V. Low | Sáez-Llorens | 2013 | (23) |
| AMR | Panama | National | 1998 | 2002 |  | Yes | Yes |  |  |  | Yes | Yes | Medium | Sáez-Llorens | 2004 | (28) |
| AMR | Panama | Panama City | 2003 | 2005 | Yes | Yes |  |  |  |  | Yes |  | V. Low | Sáez-Llorens | 2013 | (23) |
| AMR | Peru | Lima City | 2003 | 2005 | Yes | Yes |  |  |  |  | Yes |  | V. Low | Sáez-Llorens | 2013 | (23) |
| AMR | Trinidad & Tobago | Champ Fleurs | 2000 | 2007 |  |  | Yes |  |  |  |  | Yes | Medium | Tota-Maharaj | 2010 | (29) |
| AMR | USA | 41 states | 1988 | 2005 |  | Yes |  | Yes | Yes | Yes |  |  | Low | Aboagye | 2014 | (30) |
| AMR | USA | Kansas | 2001 | 2004 |  | Yes | Yes |  |  |  |  | Yes | Low | Burjonrappa | 2007 | (31) |
| AMR | USA | California | 2000 | 2005 |  | Yes |  | Yes |  | Yes | Yes |  | Low | Contopoulos-I. | 2015 | (32) |
| AMR | USA | Cinc./Nash./Roch. | 2001 | 2006 |  | Yes | Yes |  |  |  | Yes | Yes | Low | Cortese | 2009 | (33) |
| AMR | USA | National | 1998 | 2006 |  | Yes |  |  |  |  |  | Yes | V. Low | Desai | 2012 | (34) |
| AMR | USA | National | 2001 | 2005 |  | Yes | Yes |  |  |  | Yes |  | Low | Eng | 2012 | (35) |
| AMR | USA | Texas | 1996 | 2005 |  |  |  |  |  |  |  | Yes | Low | Munden | 2007 | (36) |
| AMR | USA | California | 1996 | 2007 |  | Yes | Yes |  |  |  |  | Yes | V. Low | Shekherdimian | 2011 | (37) |
| AMR | USA | 16 States/National | 1993 | 2004 |  | Yes | Yes | Yes | Yes | Yes |  | Yes | Low | Tate | 2008 | (38) |
| AMR | USA | 26 States | 2000 | 2005 |  | Yes |  | Yes |  |  | Yes |  | Low | Tate | 2016 | (39) |
| AMR | USA | 26 States | 2000 | 2005 |  | Yes | Yes | Yes |  |  |  | Yes | Medium | Yen | 2012 | (40) |
| AMR | USA | National | 2006 | 2006 |  | Yes |  |  |  |  | Yes |  | Medium | Zickafoose | 2012 | (41) |
| AMR | Venezuela | Carabobo | 1998 | 2001 |  | Yes | Yes |  |  | Yes | Yes | Yes | Low | Perez-Schael | 2003 | (42) |
| EMR | Egypt | Zagazig | 2014 | 2016 |  |  |  | Yes | Yes | Yes |  |  | Medium | Ahmed | 2015 | (43) |
| EMR | Israel (Arab) | North | 1992 | 2009 |  | Yes |  |  |  |  | Yes |  | V. Low | Muhsen | 2014 | (44) |
| EMR | Israel (Bedouin) | South | 1990 | 2004 |  | Yes | Yes | Yes | Yes | Yes | Yes | Yes | Low | Greenberg | 2008 | (45) |
| EMR | Pakistan | Karachi | 2012 | 2015 |  | Yes |  |  |  | Yes |  | Yes | Low | Yousafzai | 2017 | (46) |
| EMR | Saudi Arabia | Abha | 1993 | 2000 |  |  | Yes |  |  |  |  | Yes | Low | Al-Malki | 2005 | (47) |
| EMR | Saudi Arabia | Riyadh | 1984 | 2000 |  |  | Yes |  |  |  |  | Yes | Low | Crankson | 2003 | (48) |
| EMR | Tunisia | Monastir | 1995 | 2003 | Yes |  | Yes |  | Yes | Yes | Yes |  | Medium | Chouikha | 2009 | (49) |
| EUR | Austria | Graz | 1999 | 2006 |  |  | Yes |  |  |  |  | Yes | V. Low | Saxena | 2007 | (50) |
| EUR | Denmark | National | 1980 | 2001 |  | Yes | Yes |  |  | Yes | Yes |  | Medium | Fischer | 2004 | (51) |
| EUR | Finland | National | 2001 | 2006 |  |  | Yes |  |  |  | Yes |  | Low | Lappalainen | 2012 | (52) |
| EUR | Finland | National | 1999 | 2005 |  | Yes |  |  |  |  | Yes |  | Low | Leino | 2016 | (53) |
| EUR | Finland | National | 2000 | 2005 |  |  |  | Yes |  | Yes |  |  | ** | Vesikari (unpub) | 2018 |  |
| EUR | France | Toulouse | 2002 | 2011 |  | Yes |  |  |  | Yes |  |  | V. Low | Serayssol | 2014 | (54) |
| EUR | Germany | National | 2006 | 2007 |  | Yes | Yes |  |  |  | Yes |  | V. Low | Jenke | 2011 | (55) |
| EUR | Germany | Bavaria | 2005 | 2006 |  | Yes | Yes | Yes | Yes | Yes | Yes |  | ** | Kohl | 2010 | (56) |
| EUR | Germany | NRW and Bavaria | 2006 | 2007 |  | Yes | Yes |  |  |  | Yes |  | Low | Weiss | 2011 | (57) |
| EUR | Iceland | National | 1986 | 2010 |  | Yes |  | Yes | Yes | Yes | Yes |  | ** | Pétursdóttir | 2013 | (58) |
| EUR | Israel | Holon | 1990 | 2002 |  |  | Yes |  |  |  |  | Yes | ** | Eshed | 2003 | (59) |
| EUR | Israel (Jewish) | South | 1990 | 2004 |  | Yes | Yes | Yes | Yes | Yes | Yes | Yes | Low | Greenberg | 2008 | (45) |
| EUR | Israel (Jewish) | North | 1992 | 2009 |  | Yes |  |  |  |  | Yes |  | V. Low | Muhsen | 2014 | (44) |
| EUR | Italy | Sicily | 2003 | 2012 |  | Yes |  |  |  | Yes |  |  | Low | Costantino | 2015 | (60) |
| EUR | Italy | National | 2009 | 2014 |  | Yes |  |  |  | Yes | Yes | Yes | V. Low | Restivo | 2017 | (61) |
| EUR | Italy | National | 2002 | 2012 |  | Yes |  |  |  | Yes |  | Yes | Low | Trotta | 2016 | (62) |
| EUR | Netherlands | National | 2008 | 2012 |  | Yes |  |  |  |  | Yes |  | V. Low | Gadroen | 2017 | (63) |
| EUR | Rep. of Ireland | Waterford | 1990 | 2000 |  |  | Yes |  |  |  |  | Yes | Low | Hillal | 2002 | (64) |
| EUR | Rep. of Ireland | National | 2008 | 2009 | Yes | Yes |  |  |  |  | Yes |  | V. Low | Samad | 2013 | (65) |
| EUR | Rep. of Ireland | Not reported | 2007 | 2010 |  |  | Yes |  |  |  |  | Yes | Low | Tareen | 2011 | (66) |
| EUR | Romania | Iasi | 2009 | 2013 |  | Yes |  |  |  |  |  | Yes | Low | Tarca | 2015 | (67) |
| EUR | Russia | Vladivostok | 1994 | 2005 |  |  | Yes |  |  |  |  | Yes | Low | Shapkina | 2006 | (68) |
| EUR | Serbia | Belgrade | 1995 | 2012 | Yes |  |  |  |  |  |  | Yes | Low | Vujovic | 2014 | (69) |
| EUR | Spain | Malaga | NR | NR |  |  | Yes |  |  |  |  | Yes | Medium | Rubi | 2002 | (70) |
| EUR | Switzerland | National | 2003 | 2006 | Yes | Yes | Yes |  | Yes | Yes | Yes |  | V. Low | Buettcher | 2007 | (71) |
| EUR | Turkey | Ankara | 2002 | 2014 |  | Yes |  |  |  |  |  | Yes | Low | Guney | 2016 | (72) |
| EUR | Turkey | Sanliurfa | 2010 | 2012 |  |  |  |  |  |  |  | Yes | Low | Ocal | 2014 | (73) |
| EUR | Turkey | Ankara | 1991 | 2007 |  |  | Yes |  |  |  |  | Yes | Low | Sonmez | 2012 | (74) |
| EUR | Turkey | Ankara | 1993 | 2003 |  |  | Yes |  |  |  |  | Yes | Low | Yalcin | 2009 | (75) |
| EUR | UK | England | 2002 | 2012 |  | Yes |  | Yes | Yes | Yes |  |  | V. Low | Stowe | 2016 | (76) |
| EUR | UK | England | 2002 | 2012 |  | Yes |  |  |  |  | Yes |  | Medium | Clark | 2014 | (77) |
| EUR | UK | England | 2008 | 2009 | Yes | Yes |  |  |  | Yes | Yes |  | V. Low | Samad | 2013 | (65) |
| EUR | UK | N. Ireland | 2008 | 2009 | Yes | Yes |  |  |  |  | Yes |  | V. Low | Samad | 2013 | (65) |
| EUR | UK | Scotland | 2008 | 2009 | Yes | Yes |  |  |  |  | Yes |  | V. Low | Samad | 2013 | (65) |
| EUR | UK | Wales | 2008 | 2009 | Yes | Yes |  |  |  |  | Yes |  | V. Low | Samad | 2013 | (65) |
| EUR | UK/Rep. of Ireland | National | 2008 | 2009 | Yes | Yes | Yes |  |  |  |  | Yes | Low | Samad | 2012 | (78) |
| EUR | Uzbekistan | Bukhara | 2004 | 2008 |  | Yes | Yes | Yes |  | Yes | Yes | Yes | Low | Latipov | 2011 | (79) |
| SEA | Bangladesh | Matlab | 2004 | 2006 | Yes | Yes | Yes |  |  |  | Yes |  | Low | Zaman | 2009 | (80) |
| SEA | Bangladesh | National | 2012 | 2016 | Yes | Yes |  |  |  | Yes |  |  | Low | Satter | 2017 | (81) |
| SEA | India | Delhi | 2000 | 2003 | Yes |  | Yes |  |  |  | Yes |  | V. Low | Bahl | 2009 | (82) |
| SEA | India | Vellore | 2001 | 2004 |  | Yes | Yes |  |  |  |  | Yes | Low | Bhowmick | 2009 | (83) |
| SEA | India | Chandigargh | 2009 | 2015 | Yes | Yes |  |  | Yes |  | Yes | Yes | Low | Gupta | 2017 | (84) |
| SEA | India | Vellore | 2010 | 2013 |  |  |  |  |  |  |  | Yes | Low | Jehangir | 2014 | (85) |
| SEA | India | Delhi, Pune, Vellore | 2010 | 2013 | Yes | Yes |  |  |  |  | Yes | Yes | Low | John | 2014 | (86) |
| SEA | India | 28 hospitals | 2013 | 2015 |  | Yes |  |  |  |  | Yes |  | Medium | Mathew | 2016 | (87) |
| SEA | India | Chennai | 2012 | 2013 |  | Yes |  |  | Yes | Yes | Yes | Yes | Low | Mehendale | 2016 | (88) |
| SEA | India | Chennai | NR | NR |  |  | Yes |  |  |  |  | Yes | Low | Ramachandran | 2008 | (89) |
| SEA | India | Vellore | 1991 | 2000 |  | Yes | Yes |  |  | Yes |  | Yes | Low | Raman | 2003 | (90) |
| SEA | India | Manipal, Lucknow | 2007 | 2012 |  | Yes |  |  | Yes |  |  | Yes | Low | Singh | 2014 | (91) |
| SEA | India | Chennai | 2013 | 2016 |  | Yes |  |  | Yes | Yes |  | Yes | Low | Srinivasan | 2017 | (92) |
| SEA | India | Vellore | 2010 | 2017 |  | Yes |  | Yes | Yes | Yes |  | Yes | Low | Srinivasan | 2017 | (92) |
| SEA | Nepal | Kathmandu | 2011 | 2014 |  | Yes |  |  |  | Yes |  | Yes | V. Low | Rayamajhi | 2017 | (93) |
| SEA | Nepal | Dharan | 2004 | 2009 |  |  |  |  |  |  |  | Yes | Low | Shakya | 2011 | (94) |
| SEA | Nepal | Katmandu | 2008 | 2009 | Yes |  |  |  |  |  |  | Yes | Low | Thapa | 2012 | (95) |
| SEA | Thailand | Bangkok | 1992 | 2009 |  |  | Yes |  |  |  |  | Yes | Medium | Kruatrachue | 2011 | (96) |
| SEA | Thailand | 5 hospitals | 2001 | 2006 |  | Yes | Yes |  | Yes | Yes | Yes | Yes | Medium | Khumjui | 2009 | (97) |
| SEA | Thailand | Bangkok | 2000 | 2005 |  | Yes | Yes |  |  |  |  | Yes | Medium | Pruksananonda | 2007 | (98) |
| WPR | Australia | Brisbane | 1994 | 2004 |  |  | Yes |  |  |  |  | Yes | ** | Blanch | 2007 | (99) |
| WPR | Australia | National | 1994 | 2000 |  | Yes |  |  |  |  |  | Yes | Low | Justice | 2005 | (100) |
| WPR | Australia | Melbourne | 1995 | 2001 |  | Yes | Yes | Yes | Yes | Yes |  |  | ** | Justice | 2006 | (101) |
| WPR | Australia | Melbourne | 2001 | 2006 |  | Yes | Yes | Yes |  |  | Yes | Yes | V. Low | Lloyd-Johnsen | 2012 | (102) |
| WPR | Australia | National | 2000 | 2006 |  | Yes |  |  |  |  | Yes |  | Low | Palupi-Baroto | 2015 | (103) |
| WPR | China | Suzhou | 2007 | 2013 |  | Yes |  |  |  |  | Yes | Yes | ** | Cui | 2016 | (104) |
| WPR | China | Jinan | 2011 | 2015 |  | Yes |  |  |  |  | Yes |  | ** | Cui | 2018 | (105) |
| WPR | China | Chenzhou, Kaifeng | 2009 | 2013 |  | Yes |  | Yes |  | Yes | Yes | Yes | Low | Liu | 2018 | (106) |
| WPR | China | Shenyang | 2004 | 2009 |  |  | Yes |  |  |  |  | Yes | ** | Zhang | 2011 | (107) |
| WPR | Hong Kong | National | 1997 | 2011 |  | Yes | Yes | Yes | Yes | Yes | Yes |  | Low | Hong Kong ISG | 2007 | (108) |
| WPR | Hong Kong | Pokfulam | 1997 | 2014 |  |  |  | Yes | Yes | Yes |  |  | Low | Wong | 2015 | (109) |
| WPR | Japan | Akita | 1978 | 2002 |  |  | Yes |  | Yes | Yes | Yes |  | Low | Nakagomi | 2006 | (110) |
| WPR | Japan | Akita | 2001 | 2010 |  | Yes |  | Yes |  | Yes | Yes |  | Medium | Noguchi | 2012 | (111) |
| WPR | Japan | National | 2007 | 2008 |  | Yes | Yes | Yes | Yes | Yes | Yes | Yes | V. Low | Takeuchi | 2012 | (112) |
| WPR | Malaysia | 3 hospitals | 2000 | 2003 |  | Yes | Yes |  | Yes | Yes | Yes | Yes | Low | Giak | 2008 | (113) |
| WPR | New Zealand | National | 1998 | 2003 |  | Yes | Yes |  |  | Yes | Yes | Yes | Low | Chen | 2005 | (114) |
| WPR | New Zealand | Auckland | 1998 | 2007 |  | Yes | Yes |  |  |  |  | Yes | V. Low | Kodikara | 2010 | (115) |
| WPR | Singapore | 7 Hospitals | 2002 | 2010 | Yes | Yes |  |  |  | Yes | Yes | Yes | Low | Phua | 2013 | (116) |
| WPR | Singapore | National | 1997 | 2004 | Yes | Yes | Yes |  |  |  | Yes | Yes | Medium | Tan | 2009 | (117) |
| WPR | Singapore | 1 hospital | 2009 | 2013 |  | Yes |  |  |  |  |  | Yes | Low | Yap Shiyi | 2017 | (118) |
| WPR | South Korea | Joenbuk | 2000 | 2002 |  | Yes | Yes |  | Yes | Yes | Yes | Yes | Low | Jo | 2009 | (119) |
| WPR | Taiwan | National | 1998 | 2007 |  | Yes | Yes | Yes | Yes | Yes | Yes |  | Low | Chen | 2010 | (120) |
| WPR | Taiwan | National | 2000 | 2007 |  | Yes |  |  |  |  | Yes |  | Low | Hsiao | 2013 | (121) |
| WPR | Taiwan | Taipei | 1995 | 2010 |  |  |  |  |  |  |  | Yes | Medium | Hsu | 2012 | (122) |
| WPR | Taiwan | National | 2001 | 2005 |  | Yes |  |  |  | Yes | Yes | Yes | Low | Yen | 2017 | (123) |
| WPR | Vietnam | Hanoi | 2002 | 2004 | Yes |  | Yes |  |  | Yes | Yes | Yes | V. Low | Bines | 2006 | (124) |
| WPR | Vietnam | Hai Phong, Hue | 2013 | 2016 | Yes | Yes |  | Yes |  | Yes |  |  | Low | Trang | 2018 | (125) |
| WPR | Vietnam | Ho Chi Min City | 2009 | 2011 |  | Yes |  |  |  |  | Yes | Yes | V. Low | Van Trang | 2014 | (126) |
| WPR | Vietnam | Nha Trang | 2009 | 2011 |  | Yes |  |  | Yes | Yes | Yes |  | V. Low | Tran | 2013 | (127) |

*****Risk of bias (very low, low, medium) was assigned to each included study after assessing: a) selection bias - are participants representative of the general population?; b) baseline confounding - are characteristics of the participants reported?; c) outcome measurement - is the definition of intussusception the same for all participants?; d) blinding at outcome assessment - are data abstractors blinded to the hypothesis?; and, e) description of missing information - are drop-outs, withdrawals, missing data, or controls described?). Studies with valuable data but no full text were assigned ** to indicate that they may have a high risk of bias.

**Appendix Table 2. Studies reporting the % of intussusception hospital admissions <5 years by single year of age, before rotavirus vaccine introduction**

| **WHO region** | **Country** | **Location** | **U5MR quintile** | **Period** |  | **n** | **Cumulative % of admissions by age** | | | | |
| --- | --- | --- | --- | --- | --- | --- | --- | --- | --- | --- | --- |
|  |  |  |  | **From** | **To** |  | **1y** | **2y** | **3y** | **4y** | **5y** |
| AFR | Ghana | Accra | Very high | 2008 | 2009 | 77 | 86% | 96% | 99% | 100% | 100% |
| AFR | Kenya | National | High | 2002 | 2013 | 280 | 69% | 84% | 92% | 97% | 100% |
| AFR | Kenya | 5 hospitals | High | 2014 | 2016 | 175 | 78% | 93% | 96% | 98% | 100% |
| AFR | Nigeria | Lagos | Very high | 1995 | 2001 | 169 | 90% | 96% | 98% | 99% | 100% |
| AFR | Tanzania | Mwanza | Very high | 2010 | 2012 | 55 | 76% | 87% | 93% | 99% | 100% |
| **AFR** | **Median** |  |  |  |  |  | **78%** | **93%** | **96%** | **99%** | **100%** |
| AMR | Brazil | National | Medium | 2001 | 2006 | 943 | 80% | 90% | 95% | 98% | 100% |
| AMR | USA | 41 states | Low | 1988 | 2005 | 1171 | 62% | 83% | 95% | 100% | 100% |
| AMR | USA | 16 states/National | Low | 1994 | 2004 | 24035 | 57% | 77% | 90% | 96% | 100% |
| **AMR** | **Median** |  |  |  |  |  | **62%** | **83%** | **95%** | **98%** | **100%** |
| EMR | Egypt | Zagazig | Medium | 2014 | 2016 | 132 | 94% | 97% | 97% | 98% | 100% |
| EUR | Israel (Bedouin) | South | Medium | 1990 | 2004 | 87 | 77% | 95% | 97% | 98% | 100% |
| EMR | Tunisia | Monastir | Medium | 1995 | 2003 | 533 | 77% | 90% | 97% | 98% | 100% |
| **EMR** | **Median** |  |  |  |  |  | **77%** | **95%** | **97%** | **98%** | **100%** |
| EUR | England | National | Very low | 2002 | 2012 | 3196 | 67% | 84% | 92% | 97% | 100% |
| EUR | Germany | Bavaria | Very low | 2005 | 2011 | 752 | 27% | 56% | 75% | 90% | 100% |
| EUR | Iceland | National | Very low | 1986 | 2010 | 64 | 70% | 91% | 95% | 100% | 100% |
| EUR | Israel (Jewish) | South | Very low | 1990 | 2004 | 275 | 66% | 87% | 95% | 98% | 100% |
| EUR | Switzerland | National | Very low | 2003 | 2006 | 246 | 34% | 61% | 84% | 93% | 100% |
| **EUR** | **Median** |  |  |  |  |  | **66%** | **84%** | **92%** | **97%** | **100%** |
| SEA | India | Vellore | High | 2010 | 2017 | 219 | 75% | 92% | 95% | 98% | 100% |
| SEA | India | Chennai | High | 2012 | 2013 | 201 | 66% | 85% | 93% | 96% | 100% |
| SEA | India | Chennai | High | 2013 | 2016 | 284 | 67% | 82% | 93% | 97% | 100% |
| SEA | India | Chandigarh | High | 2009 | 2015 | 277 | 72% | 83% | 92% | 97% | 100% |
| SEA | India | Manipal + Luck. | High | 2007 | 2012 | 187 | 56% | 79% | 91% | 97% | 100% |
| SEA | Thailand | 5 hospitals | Low | 2001 | 2006 | 77 | 84% | 94% | 96% | 100% | 100% |
| **SEA** | **Median** |  |  |  |  |  | **70%** | **84%** | **93%** | **97%** | **100%** |
| WPR | Australia | Melbourne | Very low | 1995 | 2001 | 190 | 78% | 93% | 97% | 99% | 100% |
| WPR | Hong Kong | Pokfulam | Very low | 1997 | 2014 | 163 | 53% | 73% | 86% | 94% | 100% |
| WPR | Hong Kong | National | Very low | 1997 | 2011 | 520 | 62% | 82% | 92% | 96% | 100% |
| WPR | Japan | National | Very low | 2006 | 2007 | 1065 | 25% | 55% | 78% | 91% | 100% |
| WPR | Japan | Akita | Very low | 1978 | 2002 | 91 | 45% | 70% | 91% | 98% | 100% |
| WPR | Malaysia | 3 hospitals | Low | 2000 | 2003 | 62 | 74% | 90% | 95% | 98% | 100% |
| WPR | South Korea | Joenbuk | Very low | 2000 | 2002 | 408 | 53% | 83% | 93% | 97% | 100% |
| WPR | Taiwan | National | Very low | 1998 | 2013 | 10331 | 24% | 58% | 83% | 94% | 100% |
| WPR | Vietnam | Nha Trang | Medium | 2009 | 2011 | 192 | 31% | 75% | 90% | 94% | 100% |
| **WPR** | **Median** |  |  |  |  |  | **53%** | **75%** | **91%** | **96%** | **100%** |

**Appendix Table 3. Burr age distribution parameters fitted to each country and WHO region**

| **WHO region** | **Country** | **Location** | **U5MR quintile** | **n age grps** | **n** | **Parameters** | | | |  |
| --- | --- | --- | --- | --- | --- | --- | --- | --- | --- | --- |
|  |  |  |  |  |  | **Shape1** | **Shape2** | **Scale** | **RMSE** | **MAE** |
| AFR | Africa | 10 countries | Very high | 16 | 938 | 0.3 | 6.3 | 19.9 | 1% | 1% |
| AFR | Ethiopia | 6 hospitals | Very high | 56 | 207 | 0.9 | 3.3 | 32.3 | 1% | 1% |
| AFR | Ethiopia | Addis Ababa | Very high | 6 | 121 | 0.2 | 4.1 | 17.8 | 5% | 4% |
| AFR | Ghana | Accra | Very high | 10 | 77 | 0.4 | 7.8 | 24.7 | 1% | 1% |
| AFR | Ghana | 2 hospitals | Very high | 56 | 485 | 0.3 | 6.3 | 22.4 | 2% | 1% |
| AFR | Kenya | National | High | 60 | 279 | 0.3 | 4.0 | 16.3 | 5% | 4% |
| AFR | Kenya | 5 hospitals | High | 71 | 175 | 0.3 | 6.7 | 20.2 | 2% | 2% |
| AFR | Nigeria | Lagos | Very high | 27 | 169 | 3588.5 | 3.1 | 294.1 | 6% | 4% |
| AFR | Nigeria | Ibadan | Very high | 5 | 53 | 0.3 | 8.5 | 21.1 | 7% | 4% |
| AFR | Nigeria | Ile-Ife | Very high | 7 | 77 | 1.9 | 2.5 | 34.9 | 4% | 3% |
| AFR | Tanzania | 7 hospitals | Very high | 56 | 257 | 0.3 | 7.2 | 20.1 | 3% | 2% |
| AFR | Zambia | 4 hospitals | Very high | 56 | 78 | 0.5 | 5.9 | 23.4 | 4% | 3% |
| AFR | Zambia | 9 hospitals | Very high | 10 | 121 | 0.1 | 14.0 | 19.4 | 6% | 4% |
| AFR | Zimbabwe | Harare | Very high | 56 | 115 | 0.3 | 5.2 | 20.5 | 2% | 1% |
| **AFR** | Pooled |  |  |  |  | **0.4** | **4.8** | **22.2** | **1%** | **1%** |
| AMR | Brazil | National | Medium | 19 | 943 | 0.5 | 3.9 | 23.4 | 3% | 3% |
| AMR | Chile | Santiago | Low | 27 | 104 | 0.2 | 4.9 | 19.9 | 3% | 2% |
| AMR | Latin America | 11 countries | Medium | 27 | 576 | 0.2 | 7.3 | 19.6 | 4% | 3% |
| AMR | USA | 41 states | Low | 99 | 1171 | 2.0 | 3.5 | 43.0 | 2% | 1% |
| AMR | USA | 16 states /National | Low | 56 | 20027 | 0.3 | 3.6 | 25.2 | 3% | 2% |
| AMR | USA | California | Low | 56 | 1907 | 0.5 | 3.3 | 31.8 | 3% | 2% |
| AMR | Venezuela | Carabobo | Medium | 16 | 95 | 0.1 | 12.0 | 16.1 | 5% | 4% |
| **AMR** | Pooled |  |  | **27** | **23300** | **0.4** | **3.6** | **26.1** | **1%** | **0%** |
| EMR | Israel (Bedouins) | South | Medium | 16 | 83 | 0.4 | 6.1 | 26.0 | 2% | 1% |
| EMR | Egypt | Zagazig | Medium | 60 | 132 | 0.1 | 17.1 | 21.2 | 5% | 3% |
| EMR | Pakistan | Karachi | Very high | 27 | 156 | 0.4 | 5.3 | 23.2 | 2% | 1% |
| EMR | Tunisia | Monastir | Medium | 27 | 514 | 0.6 | 3.7 | 22.7 | 3% | 2% |
| **EMR** | Pooled |  |  | **27** | **880** | **0.6** | **4.1** | **25.5** | **1%** | **1%** |
| EUR | Denmark | National | Very low | 7 | 872 | 0.7 | 2.8 | 29.4 | 3% | 2% |
| EUR | England | National | Very low | 100 | 3196 | 0.4 | 3.5 | 22.9 | 2% | 1% |
| EUR | England | England | Very low | 16 | 287 | 0.5 | 2.8 | 25.2 | 2% | 2% |
| EUR | Finland | National | Very low | 27 | 280 | 0.4 | 4.9 | 24.4 | 4% | 3% |
| EUR | France | Toulouse | Very low | 14 | 276 | 8.4 | 1.7 | 295.8 | 2% | 2% |
| EUR | Germany | Bavaria | Very low | 16 | 750 | 0.6 | 2.0 | 61.5 | 4% | 3% |
| EUR | Iceland | National | Very low | 60 | 64 | 0.1 | 14.8 | 18.7 | 2% | 2% |
| EUR | Italy | National | Very low | 16 | 3088 | 0.5 | 2.2 | 47.2 | 3% | 2% |
| EUR | Italy | National | Very low | 9 | 5222 | 0.4 | 2.5 | 33.9 | 2% | 2% |
| EUR | Italy | Sicily | Very low | 16 | 340 | 0.2 | 3.9 | 24.0 | 3% | 2% |
| EUR | Israel (Jewish) | South | Very low | 16 | 273 | 0.4 | 4.6 | 29.3 | 1% | 1% |
| EUR | Switzerland | National | Very low | 16 | 246 | 0.1 | 5.7 | 18.4 | 7% | 5% |
| EUR | Uzbekistan | Bukhara | High | 13 | 80 | 0.2 | 3.3 | 16.9 | 10% | 5% |
| **EUR** | Pooled |  |  | **27** | **13539** | **0.4** | **2.8** | **29.3** | **1%** | **1%** |
| SEAR | Bangladesh | National | High | 19 | 182 | 0.3 | 7.5 | 24.4 | 4% | 3% |
| SEAR | India | Chennai | High | 7 | 207 | 1.6 | 3.2 | 43.4 | 5% | 4% |
| SEAR | India | Chennai | High | 7 | 201 | 0.8 | 3.5 | 27.4 | 6% | 5% |
| SEAR | India | Vellore | High | 8 | 137 | 0.5 | 3.6 | 25.2 | 4% | 3% |
| SEAR | India | Vellore | High | 16 | 217 | 0.3 | 6.2 | 22.6 | 2% | 2% |
| SEAR | Nepal | Kathmandu | High | 27 | 101 | 0.3 | 5.0 | 21.7 | 3% | 2% |
| SEAR | Thailand | 5 hospitals | Low | 60 | 77 | 0.5 | 4.2 | 21.2 | 2% | 2% |
| **SEA** | Pooled |  |  | **27** | **1112** | **0.6** | **4.2** | **26.4** | **0%** | **0%** |
| WPR | Australia | Melbourne | Very low | 16 | 190 | 0.5 | 4.9 | 26.7 | 1% | 1% |
| WPR | China | Chenz./, Kaif. | Medium | 104 | 2283 | 0.2 | 4.9 | 24.6 | 3% | 2% |
| WPR | Hong Kong | Pokfulam | Very low | 15 | 163 | 0.2 | 4.4 | 20.1 | 4% | 3% |
| WPR | Hong Kong | National | Very low | 60 | 520 | 0.2 | 5.8 | 21.6 | 2% | 1% |
| WPR | Japan | National | Very low | 100 | 1063 | 15.9 | 1.8 | 524.3 | 3% | 2% |
| WPR | Japan | Akita | Very low | 6 | 91 | 0.4 | 2.9 | 35.3 | 6% | 5% |
| WPR | Japan | Akita | Very low | 16 | 232 | 0.3 | 4.5 | 28.1 | 3% | 2% |
| WPR | South Korea | Joenbuk | Very low | 6 | 408 | 1.0 | 2.2 | 51.4 | 6% | 4% |
| WPR | Malaysia | 3 hospitals | Low | 7 | 62 | 0.4 | 4.2 | 25.4 | 3% | 2% |
| WPR | New Zealand | National | Very low | 38 | 305 | 0.5 | 3.1 | 28.6 | 2% | 2% |
| WPR | Singapore | 7 Hospitals | Very low | 27 | 223 | 0.1 | 5.6 | 23.7 | 5% | 3% |
| WPR | Taiwan | National | Very low | 100 | 10331 | 5180.6 | 1.9 | 8709.8 | 3% | 2% |
| WPR | Taiwan | National | Very low | 9 | 360 | 0.4 | 3.3 | 33.9 | 4% | 3% |
| WPR | Vietnam | Nha Trang | Medium | 60 | 192 | 0.8 | 2.9 | 61.0 | 2% | 2% |
| WPR | Vietnam | Hai Phong, Hue | Medium | 108 | 2916 | 0.4 | 3.4 | 43.6 | 2% | 2% |
| WPR | Vietnam | Hanoi | Medium | 68 | 779 | 0.2 | 5.3 | 26.6 | 3% | 2% |
| **WPR** | Pooled |  |  |  |  | **0.5** | **2.7** | **46.8** | **3%** | **2%** |

* The best fitting parameters for each WHO region were calculated by re-fitting Burr distributions to the pooled proportion of intussusception admissions in each week of age <5yrs. The Burr distribution (Burr type XII) has shape 1 (α), shape 2 (γ) and scale (θ), all of which must be positive values. The cumulative distribution function (cdf) is:

$$f\left( x \right)=1-\left[ 1+\left( \frac{x}{\theta} \right)^{\gamma} \right]^{-\alpha}$$

**Appendix Table 4. Median (IQR) age and cumulative proportion of intussusception hospital admissions, by age and WHO region**

| **WHO region** | **Country** | **Location** | **U5MR quintile** | **n age grps** | **n** | **IQR (weeks)** | | | **Cumulative % of intussusception hospital admissions by age** | | | | | | | | | | | | | |
| --- | --- | --- | --- | --- | --- | --- | --- | --- | --- | --- | --- | --- | --- | --- | --- | --- | --- | --- | --- | --- | --- | --- |
|  |  |  |  |  |  | **25th** | **50th** | **75th** | **6w** | **2m** | **10w** | **14w** | **15w** | **4m** | **6m** | **9m** | **12m** | **18m** | **24m** | **36m** | **48m** | **60m** |
| AFR | Africa | 10 countries | Very high | 16 | 938 | 22 | 30 | 46 | 0% | 0% | 0% | 3% | 4% | 9% | 38% | 67% | 79% | 89% | 93% | 97% | 98% | 99% |
| AFR | Ethiopia | 6 hospitals | Very high | 56 | 207 | 24 | 34 | 48 | 0% | 1% | 2% | 6% | 7% | 11% | 30% | 61% | 79% | 93% | 97% | 99% | 100% | 100% |
| AFR | Ethiopia | Addis Ababa | Very high | 6 | 121 | 22 | 36 | 73 | 0% | 1% | 2% | 7% | 9% | 14% | 34% | 54% | 65% | 77% | 82% | 88% | 91% | 93% |
| AFR | Ghana | Accra | Very high | 10 | 77 | 25 | 30 | 39 | 0% | 0% | 0% | 0% | 1% | 2% | 30% | 75% | 89% | 97% | 99% | 100% | 100% | 100% |
| AFR | Ghana | 2 hospitals | Very high | 56 | 485 | 24 | 31 | 45 | 0% | 0% | 0% | 2% | 2% | 5% | 33% | 67% | 81% | 92% | 95% | 98% | 99% | 99% |
| AFR | Kenya | National | High | 60 | 279 | 19 | 30 | 57 | 1% | 2% | 4% | 11% | 14% | 21% | 43% | 62% | 73% | 82% | 87% | 92% | 94% | 95% |
| AFR | Kenya | 5 hospitals | High | 71 | 175 | 23 | 30 | 45 | 0% | 0% | 0% | 2% | 3% | 7% | 38% | 68% | 80% | 90% | 94% | 97% | 98% | 99% |
| AFR | Nigeria | Lagos | Very high | 27 | 169 | 15 | 19 | 24 | 2% | 5% | 8% | 22% | 27% | 39% | 83% | 100% | 100% | 100% | 100% | 100% | 100% | 100% |
| AFR | Nigeria | Ibadan | Very high | 5 | 53 | 22 | 27 | 35 | 0% | 0% | 0% | 1% | 2% | 5% | 46% | 81% | 91% | 97% | 99% | 100% | 100% | 100% |
| AFR | Nigeria | Ile-Ife | Very high | 7 | 77 | 17 | 25 | 36 | 2% | 5% | 8% | 16% | 19% | 26% | 52% | 80% | 92% | 98% | 100% | 100% | 100% | 100% |
| AFR | Tanzania | 7 hospitals | Very high | 56 | 257 | 22 | 29 | 42 | 0% | 0% | 0% | 2% | 3% | 8% | 41% | 72% | 84% | 92% | 96% | 98% | 99% | 99% |
| AFR | Zambia | 4 hospitals | Very high | 56 | 78 | 23 | 29 | 38 | 0% | 0% | 0% | 2% | 3% | 7% | 39% | 76% | 89% | 96% | 98% | 99% | 100% | 100% |
| AFR | Zambia | 9 hospitals | Very high | 10 | 121 | 24 | 33 | 56 | 0% | 0% | 0% | 0% | 0% | 2% | 32% | 60% | 72% | 84% | 89% | 93% | 96% | 97% |
| AFR | Zimbabwe | Harare | Very high | 56 | 115 | 22 | 31 | 50 | 0% | 0% | 1% | 4% | 5% | 10% | 36% | 63% | 76% | 87% | 92% | 96% | 97% | 98% |
| **AFR** | Pooled |  |  |  |  | **22** | **29** | **43** | **0%** | **0%** | **1%** | **4%** | **6%** | **11%** | **39%** | **70%** | **83%** | **93%** | **96%** | **98%** | **99%** | **99%** |
| AMR | Brazil | National | Medium | 19 | 943 | 21 | 30 | 44 | 0% | 1% | 2% | 7% | 8% | 14% | 39% | 68% | 82% | 92% | 96% | 98% | 99% | 99% |
| AMR | Chile | Santiago | Low | 27 | 104 | 24 | 36 | 68 | 0% | 0% | 1% | 4% | 5% | 9% | 30% | 54% | 66% | 79% | 85% | 90% | 93% | 95% |
| AMR | Latin America | 11 countries | Medium | 27 | 576 | 23 | 32 | 52 | 0% | 0% | 0% | 2% | 3% | 6% | 34% | 62% | 75% | 86% | 91% | 95% | 96% | 97% |
| AMR | USA | 41 states | Low | 99 | 1171 | 25 | 33 | 43 | 0% | 1% | 1% | 4% | 5% | 8% | 28% | 66% | 89% | 99% | 100% | 100% | 100% | 100% |
| AMR | USA | 16 states/Nat. | Low | 56 | 20027 | 28 | 45 | 86 | 0% | 1% | 1% | 3% | 4% | 7% | 21% | 42% | 57% | 72% | 80% | 87% | 91% | 93% |
| AMR | USA | California | Low | 56 | 1907 | 29 | 43 | 68 | 0% | 1% | 1% | 3% | 4% | 7% | 20% | 44% | 62% | 80% | 88% | 94% | 96% | 98% |
| AMR | Venezuela | Carabobo | Medium | 16 | 95 | 22 | 33 | 67 | 0% | 0% | 0% | 1% | 3% | 9% | 37% | 58% | 68% | 78% | 84% | 89% | 92% | 93% |
| **AMR** | Pooled |  |  | **27** | **23300** | **27** | **41** | **69** | **0%** | **1%** | **1%** | **4%** | **5%** | **8%** | **23%** | **48%** | **63%** | **79%** | **86%** | **92%** | **95%** | **96%** |
| EMR | Israel (Bedouins) | South | Medium | 16 | 83 | 27 | 35 | 49 | 0% | 0% | 0% | 1% | 1% | 3% | 22% | 60% | 78% | 91% | 95% | 98% | 99% | 99% |
| EMR | Egypt | Zagazig | Medium | 60 | 132 | 25 | 31 | 44 | 0% | 0% | 0% | 0% | 0% | 0% | 32% | 68% | 81% | 91% | 95% | 98% | 99% | 99% |
| EMR | Pakistan | Karachi | Very high | 27 | 156 | 23 | 31 | 43 | 0% | 0% | 0% | 3% | 4% | 8% | 35% | 69% | 83% | 93% | 96% | 99% | 99% | 100% |
| EMR | Tunisia | Monastir | Medium | 27 | 514 | 20 | 28 | 41 | 0% | 2% | 3% | 9% | 11% | 17% | 44% | 72% | 84% | 93% | 97% | 99% | 99% | 100% |
| **EMR** | Pooled |  |  | **27** | **880** | **22** | **30** | **42** | **0%** | **1%** | **1%** | **5%** | **7%** | **12%** | **38%** | **71%** | **85%** | **95%** | **98%** | **99%** | **100%** | **100%** |
| EUR | Denmark | National | Very low | 7 | 872 | 23 | 35 | 57 | 1% | 2% | 3% | 8% | 10% | 14% | 31% | 56% | 71% | 86% | 92% | 96% | 98% | 99% |
| EUR | England | National | Very low | 100 | 3196 | 24 | 37 | 66 | 0% | 1% | 2% | 6% | 7% | 11% | 29% | 52% | 66% | 80% | 86% | 92% | 94% | 96% |
| EUR | England | England | Very low | 16 | 287 | 23 | 37 | 65 | 1% | 3% | 4% | 9% | 10% | 14% | 32% | 53% | 67% | 81% | 87% | 93% | 95% | 97% |
| EUR | Finland | National | Very low | 27 | 280 | 26 | 35 | 54 | 0% | 0% | 0% | 2% | 3% | 6% | 26% | 57% | 73% | 87% | 92% | 96% | 98% | 98% |
| EUR | France | Toulouse | Very low | 14 | 276 | 43 | 72 | 110 | 1% | 2% | 2% | 4% | 5% | 6% | 11% | 22% | 33% | 55% | 72% | 91% | 97% | 99% |
| EUR | Germany | Bavaria | Very low | 16 | 750 | 49 | 94 | 196 | 1% | 1% | 2% | 3% | 3% | 4% | 9% | 18% | 27% | 43% | 54% | 68% | 76% | 81% |
| EUR | Iceland | National | Very low | 60 | 64 | 25 | 37 | 73 | 0% | 0% | 0% | 0% | 0% | 2% | 29% | 53% | 65% | 77% | 82% | 88% | 91% | 93% |
| EUR | Italy | National | Very low | 16 | 3088 | 40 | 74 | 150 | 1% | 1% | 2% | 4% | 4% | 6% | 12% | 24% | 35% | 52% | 64% | 76% | 83% | 86% |
| EUR | Italy | National | Very low | 9 | 5222 | 33 | 58 | 116 | 1% | 1% | 2% | 4% | 5% | 7% | 17% | 32% | 45% | 62% | 72% | 82% | 87% | 90% |
| EUR | Italy | Sicily | Very low | 16 | 340 | 32 | 57 | 140 | 0% | 0% | 1% | 2% | 3% | 5% | 16% | 34% | 46% | 60% | 68% | 77% | 82% | 85% |
| EUR | Israel (Jewish) | South | Very low | 16 | 273 | 30 | 42 | 64 | 0% | 0% | 0% | 1% | 2% | 3% | 16% | 45% | 65% | 82% | 89% | 95% | 97% | 98% |
| EUR | Switzerland | National | Very low | 16 | 246 | 38 | 109 | 651 | 0% | 0% | 0% | 1% | 2% | 4% | 13% | 25% | 33% | 43% | 49% | 56% | 61% | 64% |
| EUR | Uzbekistan | Bukhara | High | 13 | 80 | 27 | 60 | 214 | 1% | 2% | 3% | 7% | 8% | 11% | 24% | 37% | 46% | 57% | 63% | 70% | 75% | 78% |
| **EUR** | Pooled |  |  | **27** | **13539** | **29** | **47** | **89** | **0%** | **1%** | **2%** | **5%** | **6%** | **9%** | **21%** | **40%** | **54%** | **71%** | **79%** | **87%** | **91%** | **93%** |
| SEAR | Bangladesh | National | High | 19 | 182 | 26 | 33 | 46 | 0% | 0% | 0% | 0% | 1% | 2% | 24% | 64% | 80% | 92% | 96% | 98% | 99% | 99% |
| SEAR | India | Chennai | High | 7 | 207 | 26 | 36 | 48 | 0% | 1% | 1% | 4% | 5% | 8% | 25% | 58% | 80% | 96% | 99% | 100% | 100% | 100% |
| SEAR | India | Chennai | High | 7 | 201 | 21 | 30 | 42 | 0% | 1% | 2% | 7% | 9% | 14% | 40% | 71% | 86% | 95% | 98% | 99% | 100% | 100% |
| SEAR | India | Vellore | High | 8 | 137 | 24 | 34 | 54 | 0% | 1% | 2% | 6% | 7% | 11% | 31% | 58% | 73% | 86% | 92% | 96% | 98% | 98% |
| SEAR | India | Vellore | High | 16 | 217 | 24 | 32 | 47 | 0% | 0% | 0% | 2% | 2% | 5% | 31% | 65% | 79% | 90% | 94% | 97% | 99% | 99% |
| SEAR | Nepal | Kathmandu | High | 27 | 101 | 25 | 36 | 62 | 0% | 0% | 1% | 3% | 4% | 7% | 28% | 55% | 69% | 82% | 87% | 93% | 95% | 96% |
| SEAR | Thailand | 5 hospitals | Low | 60 | 77 | 20 | 28 | 41 | 0% | 1% | 2% | 8% | 10% | 16% | 45% | 73% | 85% | 93% | 96% | 98% | 99% | 99% |
| **SEA** | Pooled |  |  | **27** | **1112** | **24** | **33** | **47** | **0%** | **1%** | **1%** | **4%** | **5%** | **8%** | **31%** | **63%** | **80%** | **92%** | **96%** | **98%** | **99%** | **99%** |
| WPR | Australia | Melbourne | Very low | 16 | 190 | 26 | 35 | 49 | 0% | 0% | 0% | 2% | 3% | 5% | 25% | 60% | 78% | 91% | 95% | 98% | 99% | 99% |
| WPR | China | Chenz./Kaif. | Medium | 104 | 2283 | 30 | 47 | 91 | 0% | 0% | 0% | 1% | 2% | 3% | 17% | 40% | 55% | 71% | 78% | 86% | 90% | 92% |
| WPR | Hong Kong | Pokfulam | Very low | 15 | 163 | 27 | 47 | 109 | 0% | 0% | 1% | 3% | 4% | 7% | 23% | 42% | 54% | 67% | 74% | 81% | 85% | 88% |
| WPR | Hong Kong | National | Very low | 60 | 520 | 27 | 41 | 79 | 0% | 0% | 0% | 1% | 2% | 5% | 23% | 47% | 61% | 75% | 81% | 88% | 91% | 93% |
| WPR | Japan | National | Very low | 100 | 1063 | 53 | 89 | 134 | 1% | 1% | 2% | 3% | 3% | 4% | 8% | 15% | 24% | 43% | 60% | 83% | 94% | 98% |
| WPR | Japan | Akita | Very low | 6 | 91 | 36 | 59 | 111 | 0% | 1% | 1% | 3% | 3% | 5% | 13% | 29% | 44% | 63% | 73% | 83% | 88% | 91% |
| WPR | Japan | Akita | Very low | 16 | 232 | 32 | 49 | 88 | 0% | 0% | 0% | 1% | 2% | 3% | 14% | 37% | 54% | 71% | 80% | 88% | 91% | 93% |
| WPR | South Korea | Joenbuk | Very low | 6 | 408 | 32 | 52 | 86 | 1% | 2% | 3% | 5% | 6% | 8% | 18% | 35% | 50% | 71% | 82% | 92% | 95% | 97% |
| WPR | Malaysia | 3 hospitals | Low | 7 | 62 | 25 | 35 | 53 | 0% | 0% | 1% | 3% | 5% | 8% | 28% | 58% | 75% | 88% | 93% | 97% | 98% | 99% |
| WPR | New Zealand | National | Very low | 38 | 305 | 26 | 40 | 66 | 0% | 1% | 2% | 6% | 7% | 10% | 26% | 49% | 65% | 81% | 88% | 94% | 96% | 97% |
| WPR | Singapore | 7 Hospitals | Very low | 27 | 223 | 35 | 63 | 169 | 0% | 0% | 0% | 1% | 1% | 2% | 12% | 30% | 43% | 57% | 65% | 74% | 78% | 82% |
| WPR | Taiwan | National | Very low | 100 | 10331 | 55 | 86 | 123 | 0% | 1% | 1% | 2% | 2% | 3% | 7% | 14% | 23% | 44% | 63% | 89% | 98% | 100% |
| WPR | Taiwan | National | Very low | 9 | 360 | 33 | 51 | 87 | 0% | 0% | 1% | 2% | 3% | 4% | 14% | 34% | 51% | 71% | 80% | 89% | 93% | 95% |
| WPR | Vietnam | Nha Trang | Medium | 60 | 192 | 47 | 70 | 107 | 0% | 0% | 0% | 1% | 1% | 2% | 6% | 17% | 31% | 57% | 74% | 88% | 94% | 96% |
| WPR | Vietnam | Hai Phong, Hue | Medium | 108 | 2916 | 43 | 67 | 115 | 0% | 0% | 0% | 1% | 1% | 2% | 6% | 20% | 35% | 58% | 71% | 84% | 89% | 92% |
| WPR | Vietnam | Hanoi | Medium | 68 | 779 | 33 | 51 | 98 | 0% | 0% | 0% | 1% | 1% | 2% | 12% | 35% | 51% | 68% | 76% | 85% | 89% | 91% |
| **WPR** | Pooled |  |  |  |  | **42** | **70** | **126** | **0%** | **1%** | **1%** | **2%** | **2%** | **3%** | **9%** | **22%** | **35%** | **56%** | **68%** | **81%** | **87%** | **90%** |

**Appendix Table 5. Studies reporting the incidence of intussusception hospital admissions among children aged <1 year and <5 years, before rotavirus vaccine introduction**

| **WHO region** | **Country** | **Location** | **U5MR quintile** | **Period** | | **n*** | **Age group** | **Rate per 100,0000 per year** | **Rate adjusted (<1yr)** | **Rate adjusted (<5yrs)** |
| --- | --- | --- | --- | --- | --- | --- | --- | --- | --- | --- |
|  |  |  |  | **From** | **To** |  |  |  |  |  |
| AFR | South Africa | 9 hospitals | High | 1998 | 2003 | 384 | <2yrs | 32.0 | 55.6 | 13.5 |
| AFR | Zambia | 9 hospitals | Very high | 2009 | 2011 | 105 | <2yrs | 7.5 | 12.9 | 3.3 |
| **AFR** | **Median** |  |  |  |  |  |  |  | **34.2** | **8.4** |
| AMR | Canada | Ontario | Very low | 2010 | 2010 | 117 | <2yrs | 18.0 | 28.2 | 8.1 |
| AMR | Chile | Santiago | Low | 2000 | 2001 | 95 | <2yrs | 33.5 | 52.0 | 15.2 |
| AMR | Panama | National | Medium | 1998 | 2002 | 111 | <3yrs | 11.0 | 23.4 | 7.2 |
| AMR | Argentina | Mendoza | Medium | 2003 | 2005 | 43 | <1yr | 105.3 | 105.3 | 33.7 |
| AMR | Brazil | Not reported | Medium | 2003 | 2005 | 19 | <1yr | 3.8 | 3.8 | 1.2 |
| AMR | Chile | Not reported | Low | 2003 | 2005 | 57 | <1yr | 47.0 | 47.0 | 14.3 |
| AMR | Colombia | Cali | Medium | 2003 | 2005 | 37 | <1yr | 37.4 | 37.4 | 11.7 |
| AMR | Costa Rica | San Jose | Low | 2003 | 2005 | 27 | <1yr | 18.9 | 18.9 | 5.7 |
| AMR | Dominican Rep. | Santo Domingo | Medium | 2003 | 2005 | 36 | <1yr | 37.8 | 37.8 | 11.9 |
| AMR | Honduras | Tegucigalpa | High | 2003 | 2005 | 40 | <1yr | 30.4 | 30.4 | 9.5 |
| AMR | Mexico | Mexico City | Medium | 2003 | 2005 | 112 | <1yr | 87.8 | 87.8 | 28.3 |
| AMR | Nicaragua | Managua | Medium | 2003 | 2005 | 10 | <1yr | 19.6 | 19.6 | 6.0 |
| AMR | Panama | Panama City | Medium | 2003 | 2005 | 112 | <1yr | 69.4 | 69.4 | 22.3 |
| AMR | Peru | Lima City | High | 2003 | 2005 | 10 | <1yr | 25.1 | 25.1 | 8.1 |
| AMR | USA | National | Low | 2001 | 2005 | 22 | <1yr | 33.0 | 33.0 | 9.9 |
| AMR | USA | National | Low | 2006 | 2006 | 1548 | <1yr | 36.5 | 36.5 | 10.9 |
| AMR | USA | California | Low | 2000 | 2005 | 1187 | <1yr | 37.0 | 37.0 | 11.1 |
| AMR | USA | Cinc./Nash./Roch. | Low | 2001 | 2006 | 156 | <1yr | 49.3 | 49.3 | 14.7 |
| AMR | USA | 26 States | Low | 2000 | 2005 | 15231 | <1yr | 35.9 | 35.9 | 10.7 |
| AMR | Venezuela | Carabobo | Medium | 1998 | 2001 | 67 | <1yr | 35.0 | 35.0 | 11.0 |
| **AMR** | **Median** |  |  |  |  |  |  |  | **36.2** | **11.0** |
| EMR | Tunisia | Monastir | Medium | 1984 | 2003 | 533 | <5yrs | 13.0 | 51.1 | 13.0 |
| EMR | Israel (Arab) | North | Medium | 1992 | 2009 | 76 | <5yrs | 23.2 | 95.2 | 23.2 |
| EMR | Israel (Bedouin) | South | Medium | 1990 | 2004 | 75 | <5yrs | 18.9 | 77.5 | 18.9 |
| **EMR** | **Median** |  |  |  |  |  |  |  | **77.5** | **18.9** |
| EUR | Denmark | National | Very low | 1980 | 2001 | 1814 | <5yrs | 27.2 | 90.9 | 27.2 |
| EUR | Finland | National | Very low | 2001 | 2006 | 53 | <1yr | 20.0 | 20.0 | 7.0 |
| EUR | Finland | National | Very low | 1999 | 2005 | 52 | <1yr | 12.1 | 12.1 | 4.2 |
| EUR | Germany | National | Very low | 2006 | 2007 | 1200 | <2yrs | 51.5 | 75.6 | 26.2 |
| EUR | Germany | NRW and Bavaria | Very low | 2006 | 2007 | 169 | <1yr | 61.7 | 61.7 | 23.0 |
| EUR | Germany | Bavaria | Very low | 2005 | 2006 | 518 | <1yr | 72.0 | 72.0 | 26.8 |
| EUR | Iceland | National | Very low | 1986 | 2010 | 42 | <1yr | 40.0 | 40.0 | 13.6 |
| EUR | Ireland | National | Very low | 2008 | 2009 | 21 | <1yr | 24.8 | 24.8 | 8.6 |
| EUR | Israel (Jewish) | South | Very low | 1990 | 2004 | 241 | <5yrs | 49.3 | 137.9 | 49.3 |
| EUR | Israel (Jewish) | North | Very low | 1992 | 2009 | 114 | <5yrs | 36.1 | 101.0 | 36.1 |
| EUR | Italy | National | Very low | 2009 | 2014 | 3088 | <5yrs | 20.2 | 64.2 | 20.2 |
| EUR | Netherlands | National | Very low | 2008 | 2012 | 15 | <3yrs | 21.3 | 42.4 | 14.6 |
| EUR | Switzerland | National | Very low | 2003 | 2006 | 294 | <3yrs | 26.0 | 51.8 | 18.0 |
| EUR | UK | England | Very low | 2002 | 2012 | 2692 | <2yrs | 18.0 | 27.0 | 8.8 |
| EUR | UK | England | Very low | 2008 | 2009 | 190 | <1yr | 24.2 | 24.2 | 8.5 |
| EUR | UK | N. Ireland | Very low | 2008 | 2009 | 12 | <1yr | 40.6 | 40.6 | 14.3 |
| EUR | UK | Scotland | Very low | 2008 | 2009 | 20 | <1yr | 28.7 | 28.7 | 10.1 |
| EUR | UK | Wales | Very low | 2008 | 2009 | 7 | <1yr | 16.9 | 16.9 | 5.9 |
| EUR | Uzbekistan | Bukhara | High | 2004 | 2008 | 67 | <2yrs | 23.0 | 34.3 | 11.5 |
| **EUR** | **Median** |  |  |  |  |  |  |  | **40.6** | **14.3** |
| SEA | Bangladesh | Matlab | High | 2004 | 2006 | 3 | <2yrs | 9.4 | 15.6 | 3.9 |
| SEA | India | Delhi | High | 2000 | 2003 | 5 | <1yr | 17.7 | 17.7 | 4.3 |
| SEA | India | Chandigargh | High | 2009 | 2015 | 277 | <5yrs | 5.0 | 20.7 | 5.0 |
| SEA | India | Delhi/Pune/Vellore | High | 2010 | 2013 | 3 | <2yrs | 71.0 | 120.1 | 28.9 |
| SEA | India | 28 hospitals | High | 2013 | 2015 | 98 | <5yrs | 46.8 | 194.1 | 46.8 |
| SEA | India | Chennai | High | 2012 | 2013 | 201 | <5yrs | 61.0 | 253.0 | 61.0 |
| SEA | Thailand | 5 hospitals | Low | 2001 | 2006 | 112 | <5yrs | 8.1 | 33.6 | 8.1 |
| **SEA** | **Median** |  |  |  |  |  |  |  | **33.6** | **8.1** |
| WPR | Australia | Melbourne | Very low | 2001 | 2006 | 135 | <2yrs | 19.9 | 23.0 | 11.4 |
| WPR | Australia | National | Very low | 2000 | 2006 | 1650 | <2yrs | 45.1 | 52.1 | 25.9 |
| WPR | China | Suzhou | Medium | 2007 | 2013 | 594 | <2yrs | 57.3 | 65.3 | 33.8 |
| WPR | China | Jinan | Medium | 2011 | 2015 | 93 | <2yrs | 86.3 | 98.3 | 50.9 |
| WPR | China | Suzhou | Medium | 2007 | 2013 | 1715 | <2yrs | 112.9 | 128.7 | 66.6 |
| WPR | Hong Kong | National | Very low | 1997 | 2003 | 531 | <5yrs | 38.4 | 82.4 | 38.4 |
| WPR | Taiwan | National | Very low | 1998 | 2007 | 8217 | <5yrs | 45.5 | 78.6 | 45.5 |
| WPR | Taiwan | National | Very low | 2000 | 2007 | 5721 | <5yrs | 56.6 | 97.7 | 56.6 |
| WPR | Taiwan | 26 States | Very low | 2001 | 2005 | 189 | <1yr | 82.2 | 82.2 | 52.7 |
| WPR | Japan | National | Very low | 2007 | 2008 | 2427 | <1yr | 185.0 | 185.0 | 103.2 |
| WPR | Japan | North | Very low | 1978 | 2002 | 91 | <5yrs | 77.8 | 154.5 | 77.8 |
| WPR | Japan | Akita | Very low | 2001 | 2010 | 122 | <1yr | 158.0 | 158.0 | 88.1 |
| WPR | Malaysia | 3 hospitals | Low | 2000 | 2003 | 62 | <5yrs | 4.8 | 9.0 | 4.8 |
| WPR | New Zealand | National | Very low | 1998 | 2003 | 277 | <3yrs | 30.0 | 45.0 | 21.7 |
| WPR | South Korea | Jeonbuk | Very low | 2000 | 2002 | 408 | <5yrs | 106.4 | 208.6 | 106.4 |
| WPR | Singapore | National | Very low | 1997 | 2004 | 217 | <2yrs | 32.4 | 36.9 | 18.8 |
| WPR | Singapore | 7 Hospitals | Very low | 2002 | 2010 | 167 | <2yrs | 26.1 | 29.8 | 15.2 |
| WPR | Vietnam | Hanoi | Medium | 2002 | 2004 | 533 | <1yr | 302.0 | 302.0 | 172.4 |
| WPR | Vietnam | Ho Chi Min City | Medium | 2009 | 2011 | 869 | <1yr | 287.0 | 287.0 | 163.9 |
| WPR | Vietnam | Nha Trang | Medium | 2009 | 2011 | 187 | <5yrs | 196.1 | 380.2 | 196.1 |
| **WPR** | **Median** |  |  |  |  |  |  |  | **90.1** | **51.8** |

* some numerators were derived from incidence and denominator data

**Appendix Table 6. Case fatality ratios for intussusception hospital admissions among children aged <5 years**

| **WHO region** | **Country** | **Location** | **U5MR quintile** | **Age group** | **CFR (age unadjusted), meta-analysis** | | | | | **% CFR adjusted to <5 years** |
| --- | --- | --- | --- | --- | --- | --- | --- | --- | --- | --- |
|  |  |  |  |  | **Deaths** | **Cases** | **% CFR** | **L95** | **U95** |  |
| AFR | Africa | 10 countries | Very high | <1yr | 108 | 863 | 12.51% | 10.38% | 14.91% | 10.77% |
| AFR | Ethiopia | Addis Ababa | Very high | <5yrs+ | 6 | 130 | 4.62% | 1.71% | 9.78% | 4.62% |
| AFR | Ethiopia | 6 hospitals | Very high | <1yr | 19 | 155 | 12.26% | 7.54% | 18.48% | 10.53% |
| AFR | Ghana | Kumasi | Very high | <5yrs+ | 1 | 44 | 2.27% | 0.06% | 12.02% | 2.27% |
| AFR | Ghana | 2 hospitals | Very high | <1yr | 9 | 360 | 2.50% | 1.15% | 4.69% | 2.15% |
| AFR | Kenya | Eldoret | High | <5yrs+ | 5 | 36 | 13.89% | 4.67% | 29.50% | 13.89% |
| AFR | Kenya | National | High | <5yrs | 18 | 280 | 6.43% | 3.85% | 9.97% | 6.43% |
| AFR | Kenya | Bomet | High | <5yrs+ | 3 | 30 | 10.00% | 2.11% | 26.53% | 10.00% |
| AFR | Kenya | 5 hospitals | High | <1yr | 20 | 126 | 15.87% | 9.97% | 23.44% | 13.58% |
| AFR | Malawi | 4 hospitals | Very high | <1yr | 4 | 26 | 15.38% | 4.36% | 34.87% | 13.09% |
| AFR | Nigeria | Lagos | Very high | <5yrs+ | 21 | 174 | 12.07% | 7.63% | 17.86% | 12.07% |
| AFR | Nigeria | Enugu | Very high | <5yrs+ | 7 | 87 | 8.05% | 3.30% | 15.88% | 8.05% |
| AFR | Nigeria | Enugu | Very high | <2yrs | 0 | 20 | 0.00% | 0.00% | 16.84% | 0.00% |
| AFR | Nigeria | Ile-Ife | Very high | <5yrs+ | 12 | 78 | 15.38% | 8.21% | 25.33% | 15.38% |
| AFR | Nigeria | Enugu | Very high | <5yrs+ | 2 | 58 | 3.45% | 0.42% | 11.91% | 3.45% |
| AFR | Nigeria | Ibadan | Very high | <5yrs+ | 3 | 55 | 5.45% | 1.14% | 15.12% | 5.45% |
| AFR | Rwanda | Kigali | Very high | <5yrs+ | 17 | 60 | 28.33% | 17.45% | 41.44% | 28.33% |
| AFR | South Africa | Not reported | High | <5yrs+ | 10 | 106 | 9.43% | 4.62% | 16.67% | 9.43% |
| AFR | South Africa | 9 hospitals | High | <5yrs+ | 9 | 423 | 2.13% | 0.98% | 4.00% | 2.13% |
| AFR | South Africa | Bloemfontein | High | <5yrs+ | 0 | 35 | 0.00% | 0.00% | 10.00% | 0.00% |
| AFR | South Africa | Johannesburg | High | <3yrs | 9 | 99 | 9.09% | 4.24% | 16.56% | 9.01% |
| AFR | Tanzania | Dar es Salaam | Very high | <5yrs+ | 7 | 28 | 25.00% | 10.69% | 44.87% | 25.00% |
| AFR | Tanzania | Mwanza | Very high | <5yrs+ | 8 | 56 | 14.29% | 6.38% | 26.22% | 14.29% |
| AFR | Tanzania | 7 hospitals | Very high | <1yr | 57 | 182 | 31.32% | 24.66% | 38.60% | 26.85% |
| AFR | Zambia | 9 hospitals | Very high | <2yrs | 31 | 92 | 33.70% | 24.17% | 44.30% | 32.83% |
| AFR | Zambia | 4 hospitals | Very high | <1yr | 13 | 54 | 24.07% | 13.49% | 37.64% | 20.69% |
| AFR | Zimbabwe | Harare | Very high | <1yr | 8 | 82 | 9.76% | 4.31% | 18.32% | 8.37% |
|  | **Africa** |  |  |  | **407** | **3739** | **11.50%** | **7.24%** | **17.78%** | **10.08%** |
| AMR | Canada | Toronto | Very low | <5yrs+ | 0 | 41 | 0.00% | 0.00% | 8.60% | 0.00% |
| AMR | Chile | Santiago | Low | <2yrs | 0 | 86 | 0.00% | 0.00% | 4.20% | 0.00% |
| AMR | Latin America | 11 countries | Medium | <2yrs | 13 | 476 | 2.73% | 1.46% | 4.62% | 2.48% |
| AMR | Panama | National | Medium | <3yrs | 1 | 111 | 0.90% | 0.02% | 4.92% | 0.87% |
| AMR | Trinidad & Tobago | Champ Fleurs | High | <3yrs | 0 | 65 | 0.00% | 0.00% | 5.52% | 0.00% |
| AMR | USA | Texas | Low | <5yrs+ | 1 | 35 | 2.86% | 0.07% | 14.92% | 2.86% |
| AMR | USA | Kansas | Low | <5yrs+ | 0 | 26 | 0.00% | 0.00% | 13.23% | 0.00% |
| AMR | USA | 16 States/National | Low | <1yr | 14 | 3,463 | 0.40% | 0.22% | 0.68% | 0.27% |
| AMR | USA | Cinc./Nash./Roch. | Low | <1yr | 2 | 156 | 1.28% | 0.16% | 4.55% | 0.86% |
| AMR | USA | California | Low | <5yrs+ | 0 | 188 | 0.00% | 0.00% | 1.94% | 0.00% |
| AMR | USA | 26 States | Low | <1yr | 6 | 6,502 | 0.09% | 0.03% | 0.20% | 0.06% |
| AMR | USA | National | Low | <1yr | 80 | 36,400 | 0.22% | 0.17% | 0.27% | 0.15% |
| AMR | Venezuela | Carabobo | Medium | <1yr | 0 | 67 | 0.00% | 0.00% | 5.36% | 0.00% |
|  | **Americas** |  |  |  | **117** | **47616** | **0.41%** | **0.11%** | **1.54%** | **0.17%** |
| EMR | Israel | Holon | Very low | <5yrs | 0 | 148 | 0.00% | 0.00% | 2.46% | 0.00% |
| EMR | Pakistan | Karachi | Very high | <2yrs | 3 | 149 | 2.01% | 0.42% | 5.77% | 1.97% |
| EMR | Saudi Arabia | Riyadh | Medium | <3yrs | 0 | 37 | 0.00% | 0.00% | 9.49% | 0.00% |
| EMR | Saudi Arabia | Abha | Medium | <5yrs+ | 0 | 34 | 0.00% | 0.00% | 10.28% | 0.00% |
|  | **Eastern Mediterranean** | |  |  | **3** | **368** | **0.46%** | **0.02%** | **8.74%** | **0.81%** |
| EUR | Austria | Graz | Very low | <5yrs+ | 0 | 111 | 0.00% | 0.00% | 3.27% | 0.00% |
| EUR | Israel (Bedouin) | South | Very low | <5yrs | 0 | 75 | 0.00% | 0.00% | 4.80% | 0.00% |
| EUR | Israel (Jewish) | South | Very low | <5yrs | 0 | 241 | 0.00% | 0.00% | 1.52% | 0.00% |
| EUR | Italy | National | Very low | <5yrs | 6 | 5,222 | 0.11% | 0.04% | 0.25% | 0.11% |
| EUR | Italy | National | Very low | <5yrs | 2 | 3,088 | 0.06% | 0.01% | 0.23% | 0.06% |
| EUR | Rep. of Ireland | Waterford | Very low | <2yrs | 0 | 24 | 0.00% | 0.00% | 14.25% | 0.00% |
| EUR | Rep. of Ireland | Not reported | Very low | <5yrs+ | 0 | 256 | 0.00% | 0.00% | 1.43% | 0.00% |
| EUR | Romania | Iasi | Low | <5yrs+ | 3 | 45 | 6.67% | 1.40% | 18.27% | 6.67% |
| EUR | Russia | Vladivostok | Low | <5yrs+ | 0 | 280 | 0.00% | 0.00% | 1.31% | 0.00% |
| EUR | Serbia | Belgrade | Low | <5yrs+ | 0 | 107 | 0.00% | 0.00% | 3.39% | 0.00% |
| EUR | Spain | Malaga | Very low | <5yrs+ | 1 | 151 | 0.66% | 0.02% | 3.63% | 0.66% |
| EUR | Turkey | Ankara | Medium | <5yrs+ | 0 | 179 | 0.00% | 0.00% | 2.04% | 0.00% |
| EUR | Turkey | Ankara | Medium | <5yrs+ | 1 | 105 | 0.95% | 0.02% | 5.19% | 0.95% |
| EUR | Turkey | Sanliurfa | Medium | <5yrs+ | 0 | 72 | 0.00% | 0.00% | 4.99% | 0.00% |
| EUR | Turkey | Ankara | Medium | <5yrs+ | 0 | 81 | 0.00% | 0.00% | 4.45% | 0.00% |
| EUR | UK/Rep. of Ireland | National | Very low | <1yr | 1 | 261 | 0.38% | 0.01% | 2.12% | 0.23% |
| EUR | Uzbekistan | Bukhara | High | <2yrs | 4 | 67 | 5.97% | 1.65% | 14.59% | 5.14% |
|  | **Europe** |  |  |  | **18** | **10,365** | **0.20%** | **0.05%** | **0.89%** | **0.17%** |
| SEA | India | Vellore | High | <5yrs | 1 | 137 | 0.73% | 0.02% | 4.00% | 0.73% |
| SEA | India | Chennai | High | <5yrs+ | 0 | 179 | 0.00% | 0.00% | 2.04% | 0.00% |
| SEA | India | Vellore | High | <5yrs | 0 | 31 | 0.00% | 0.00% | 11.22% | 0.00% |
| SEA | India | Delhi, Pune, Vellore | High | <2yrs | 0 | 3 | 0.00% | 0.00% | 70.76% | 0.00% |
| SEA | India | Vellore | High | <2yrs | 0 | 59 | 0.00% | 0.00% | 6.06% | 0.00% |
| SEA | India | Manipal, Lucknow | High | <5yrs | 0 | 187 | 0.00% | 0.00% | 1.95% | 0.00% |
| SEA | India | Chennai | High | <5yrs | 0 | 201 | 0.00% | 0.00% | 1.82% | 0.00% |
| SEA | India | Chennai | High | <5yrs | 3 | 207 | 1.45% | 0.30% | 4.18% | 1.45% |
| SEA | India | Vellore | High | <5yrs | 0 | 77 | 0.00% | 0.00% | 4.68% | 0.00% |
| SEA | India | Chandigargh | High | <5yrs | 0 | 277 | 0.00% | 0.00% | 1.32% | 0.00% |
| SEA | Nepal | Dharan | High | <5yrs+ | 3 | 47 | 6.38% | 1.34% | 17.54% | 6.38% |
| SEA | Nepal | Kathmandu | High | <5yrs+ | 0 | 34 | 0.00% | 0.00% | 10.28% | 0.00% |
| SEA | Nepal | Kathmandu | High | <2yrs | 1 | 85 | 1.18% | 0.03% | 6.38% | 1.14% |
| SEA | Thailand | Bangkok | Low | <5yrs+ | 0 | 94 | 0.00% | 0.00% | 3.85% | 0.00% |
| SEA | Thailand | 5 hospitals | Low | <5yrs | 0 | 112 | 0.00% | 0.00% | 3.24% | 0.00% |
| SEA | Thailand | Bangkok | Low | <5yrs+ | 0 | 737 | 0.00% | 0.00% | 0.50% | 0.00% |
|  | **South East Asia** |  |  |  | **8** | **2467** | **0.27%** | **0.03%** | **2.48%** | **0.32%** |
| WPR | Australia | National | Very low | <1yr | 1 | 1,794 | 0.06% | 0.00% | 0.31% | 0.02% |
| WPR | Australia | Brisbane | Very low | <5yrs+ | 0 | 141 | 0.00% | 0.00% | 2.58% | 0.00% |
| WPR | Australia | Melbourne | Very low | <2yrs | 0 | 135 | 0.00% | 0.00% | 2.70% | 0.00% |
| WPR | China | Shenyang | Medium | <5yrs+ | 0 | 56 | 0.00% | 0.00% | 6.38% | 0.00% |
| WPR | China | Suzhou | Medium | <2yrs | 0 | 594 | 0.00% | 0.00% | 0.62% | 0.00% |
| WPR | China | Chenzhou, Kaifeng | Medium | <2yrs | 0 | 1,714 | 0.00% | 0.00% | 0.21% | 0.00% |
| WPR | Japan | National | Very low | <5yrs+ | 2 | 2,427 | 0.08% | 0.01% | 0.30% | 0.08% |
| WPR | Malaysia | 3 hospitals | Low | <5yrs | 0 | 62 | 0.00% | 0.00% | 5.78% | 0.00% |
| WPR | New Zealand | National | Very low | <3yrs | 0 | 277 | 0.00% | 0.00% | 1.32% | 0.00% |
| WPR | New Zealand | Auckland | Very low | <5yrs+ | 0 | 189 | 0.00% | 0.00% | 1.93% | 0.00% |
| WPR | Singapore | National | Very low | <2yrs | 0 | 217 | 0.00% | 0.00% | 1.69% | 0.00% |
| WPR | Singapore | 7 hospitals | Very low | <2yrs | 1 | 167 | 0.60% | 0.02% | 3.29% | 0.47% |
| WPR | Singapore | 1 hospital | Very low | <5yrs+ | 0 | 391 | 0.00% | 0.00% | 0.94% | 0.00% |
| WPR | South Korea | Joenbuk | Very low | <5yrs | 1 | 408 | 0.25% | 0.01% | 1.36% | 0.25% |
| WPR | Taiwan | Taipei | Very low | <5yrs+ | 0 | 686 | 0.00% | 0.00% | 0.54% | 0.00% |
| WPR | Taiwan | National | Very low | <1yr | 1 | 946 | 0.11% | 0.00% | 0.59% | 0.05% |
| WPR | Vietnam | Hanoi | Medium | <2yrs | 0 | 533 | 0.00% | 0.00% | 0.69% | 0.00% |
| WPR | Vietnam | Ho Chi Min City | Medium | <1yr | 0 | 869 | 0.00% | 0.00% | 0.42% | 0.00% |
|  | **Western Pacific** |  |  |  | **6** | **11606** | **0.05%** | **0.02%** | **0.12%** | **0.03%** |

**List of references**

1. Steele AD, Patel M, Cunliffe NA, Bresee JS, Borgstein E, Parashar UD. Workshop on intussusception in African countries--meeting report. Vaccine. 2012;30 Suppl 1:A185-9.

2. Gadisa A, Tadesse A, Hailemariam B. Patterns and Seasonal Variation of Intussusception in Children: A Retrospective Analysis of Cases Operated in a Tertiary Hospital in Ethiopia. Ethiop Med J. 2016;54(1):9-15.

3. Tate JE, Mwenda JM, Armah G, Jani B, Omore R, Ademe A, et al. Evaluation of Intussusception after Monovalent Rotavirus Vaccination in Africa. N Engl J Med. 2018;378(16):1521-8.

4. Abantanga FA, Amoah M, Adeyinka AO, Nimako B, Yankey KP. Pneumatic reduction of intussusception in children at the Komfo Anokye Hospital, Kumasi, Ghana. East Afr Med J. 2008;85(11):550-5.

5. Enweronu-Laryea CC, Sagoe KW, Glover-Addy H, Asmah RH, Mingle JA, Armah GE. Prevalence of severe acute rotavirus gastroenteritis and intussusceptions in Ghanaian children under 5 years of age. J Infect Dev Ctries. 2012;6(2):148-55.

6. Kuremu RT. Childhood intussusception at the Moi teaching and Referral Hospital Eldoret: management challenges in a rural setting. East Afr Med J. 2004;81(9):443-6.

7. Omore R, Osawa F, Musia J, Rha B, Ismail A, Kiulia NM, et al. Intussusception Cases Among Children Admitted to Referral Hospitals in Kenya, 2002-2013: Implications for Monitoring Postlicensure Safety of Rotavirus Vaccines in Africa. J Pediatric Infect Dis Soc. 2016;5(4):465-9.

8. Ooko PB, Wambua P, Oloo M, Odera A, Topazian HM, White R. The Spectrum of Paediatric Intestinal Obstruction in Kenya. Pan Afr Med J. 2016;24:43.

9. Bode CO. Presentation and management outcome of childhood intussusception in Lagos: a prospective study. Afr J Paediatr Surg. 2008;5(1):24-8.

10. Ekenze SO, Mgbor SO, Okwesili OR. Routine surgical intervention for childhood intussusception in a developing country. Ann Afr Med. 2010;9(1):27-30.

11. Ekenze SO, Mgbor SO. Childhood intussusception: the implications of delayed presentation. Afr J Paediatr Surg. 2011;8(1):15-8.

12. Ekenze SO, Chukwubuike KE, Ezomike UO, Okere PC, Onuh AC. Pediatric Intussusception and Interventional Radiology in a Developing Country: Experienceand Challenges of Ultrasound Saline Reduction Complementary to Primary Surgery. Available at: <http://www.internationalsurgery.org/doi/pdf/10.9738/INTSURG-D-14-00190.1?code=icsu-site> [accessed 29th July 2018]. International Surgery. 2015;100:1301–7.

13. Ogundoyin OO, Olulana DI, Lawal TA. Childhood intussusception: Impact of delay in presentation in a developing country. Afr J Paediatr Surg. 2016;13(4):166-9.

14. Talabi AO, Sowande OA, Etonyeaku CA, Adejuyigbe O. Childhood intussusception in Ile-ife: what has changed? Afr J Paediatr Surg. 2013;10(3):239-42.

15. Ngendahayo E, Bonane A, Ntakiyiruta G, Munyanshongore A, Muganga N, Bikoroti J, et al. Preparing for safety monitoring after rotavirus vaccine implementation: a retrospective review of intussusception cases among children at a large teaching hospital in Rwanda, 2009-2012. Pediatr Infect Dis J. 2014;33 Suppl 1:S99-S103.

16. Carapinha C, Truter M, Bentley A, Welthagen A, Loveland J. Factors determining clinical outcomes in intussusception in the developing world: Experience from Johannesburg, South Africa. S Afr Med J. 2016;106(2):177-80.

17. Moore SW, Kirsten M, Muller EW, Numanoglu A, Chitnis M, Le Grange E, et al. Retrospective surveillance of intussusception in South Africa, 1998-2003. J Infect Dis. 2010;202 Suppl:S156-61.

18. Venter JA, le Grange SM, Otto SF, Joubert G. An audit of paediatric intussusception radiological reduction at the Bloemfontein Academic Hospital Complex, Free State, South Africa. Available at: <http://www.sajch.org.za/index.php/SAJCH/article/view/531/400> [accessed 29th July 2018]. South African Journal of Child Health 2013;7(2).

19. Wiersma R, Hadley GP. Minimizing surgery in complicated intussusceptions in the Third World. Pediatr Surg Int. 2004;20(3):215-7.

20. Carneiro PM, Kisusi DM. Intussusception in children seen at Muhimbili National Hospital, Dar es Salaam. East Afr Med J. 2004;81(9):439-42.

21. Chalya PL, Kayange NM, Chandika AB. Childhood intussusceptions at a tertiary care hospital in northwestern Tanzania: a diagnostic and therapeutic challenge in resource-limited setting. Ital J Pediatr. 2014;40(1):28.

22. Mpabalwani EM, Chitambala P, Chibumbya JN, Matapo B, Mutambo H, Mwenda JM, et al. Intussusception incidence rates in 9 Zambian hospitals, 2007-2011: prerotavirus vaccine introduction. Pediatr Infect Dis J. 2014;33 Suppl 1:S94-8.

23. Saez-Llorens X, Velazquez FR, Lopez P, Espinoza F, Linhares AC, Abate H, et al. A multi-country study of intussusception in children under 2 years of age in Latin America: analysis of prospective surveillance data. BMC Gastroenterol. 2013;13:95.

24. Teles E, Moscovici L, Monteiro RA, Alves D, Laprega MR, Bellissimo-Rodrigues F. The effectiveness of a rotavirus vaccine in preventing hospitalizations and deaths presumably due to acute infectious diarrhea in Brazilian children: a quasi-experimental study. Rev Soc Bras Med Trop. 2015;48(2):129-35.

25. Bailey KA, Wales PW, Gerstle JT. Laparoscopic versus open reduction of intussusception in children: a single-institution comparative experience. J Pediatr Surg. 2007;42(5):845-8.

26. Ducharme R, Benchimol EI, Deeks SL, Hawken S, Fergusson DA, Wilson K. Validation of diagnostic codes for intussusception and quantification of childhood intussusception incidence in Ontario, Canada: a population-based study. J Pediatr. 2013;163(4):1073-9 e3.

27. O'Ryan M, Lucero Y, Pena A, Valenzuela MT. Two year review of intestinal intussusception in six large public hospitals of Santiago, Chile. Pediatr Infect Dis J. 2003;22(8):717-21.

28. Saez-Llorens X, Guevara JN. Intussusception and rotavirus vaccines: what is the background risk? Pediatr Infect Dis J. 2004;23(4):363-5.

29. Tota-Maharaj R, Rampersad B, Indalsingh R. Barium enema reduction of intussusception in a developing country. West Indian Med J. 2010;59(5):535-9.

30. Aboagye J, Goldstein SD, Salazar JH, Papandria D, Okoye MT, Al-Omar K, et al. Age at presentation of common pediatric surgical conditions: Reexamining dogma. J Pediatr Surg. 2014;49(6):995-9.

31. Burjonrappa SC. Laparoscopic reduction of intussusception: an evolving therapeutic option. JSLS. 2007;11(2):235-7.

32. Contopoulos-Ioannidis DG, Halpern MS, Maldonado Y. Trends in Hospitalizations for Intussusception in California in Relationship to the Introduction of New Rotavirus Vaccines, 1985-2010. Pediatr Infect Dis J. 2015;34(7):712-7.

33. Cortese MM, Staat MA, Weinberg GA, Edwards K, Rice MA, Szilagyi PG, et al. Underestimates of intussusception rates among US infants based on inpatient discharge data: implications for monitoring the safety of rotavirus vaccines. J Infect Dis. 2009;200 Suppl 1:S264-70.

34. Desai R, Curns AT, Patel MM, Parashar UD. Trends in intussusception-associated deaths among US infants from 1979-2007. J Pediatr. 2012;160(3):456-60.

35. Eng PM, Mast TC, Loughlin J, Clifford CR, Wong J, Seeger JD. Incidence of intussusception among infants in a large commercially insured population in the United States. Pediatr Infect Dis J. 2012;31(3):287-91.

36. Munden MM, Bruzzi JF, Coley BD, Munden RF. Sonography of pediatric small-bowel intussusception: differentiating surgical from nonsurgical cases. AJR Am J Roentgenol. 2007;188(1):275-9.

37. Shekherdimian S, Lee SL. Management of pediatric intussusception in general hospitals: diagnosis, treatment, and differences based on age. World J Pediatr. 2011;7(1):70-3.

38. Tate JE, Simonsen L, Viboud C, Steiner C, Patel MM, Curns AT, et al. Trends in intussusception hospitalizations among US infants, 1993-2004: implications for monitoring the safety of the new rotavirus vaccination program. Pediatrics. 2008;121(5):e1125-32.

39. Tate JE, Yen C, Steiner CA, Cortese MM, Parashar UD. Intussusception Rates Before and After the Introduction of Rotavirus Vaccine. Pediatrics. 2016;138(3).

40. Yen C, Tate JE, Steiner CA, Cortese MM, Patel MM, Parashar UD. Trends in intussusception hospitalizations among US infants before and after implementation of the rotavirus vaccination program, 2000-2009. J Infect Dis. 2012;206(1):41-8.

41. Zickafoose JS, Benneyworth BD, Riebschleger MP, Espinosa CM, Davis MM. Hospitalizations for intussusception before and after the reintroduction of rotavirus vaccine in the United States. Arch Pediatr Adolesc Med. 2012;166(4):350-5.

42. Perez-Schael I, Escalona M, Salinas B, Materan M, Perez ME, Gonzalez G. Intussusception-associated hospitalization among Venezuelan infants during 1998 through 2001: anticipating rotavirus vaccines. Pediatr Infect Dis J. 2003;22(3):234-9.

43. Ahmed HM, Ahmed O, Ahmed RK. Adding a custom made pressure release valve during air enema for intussusception: A new technique. Afr J Paediatr Surg. 2015;12(4):232-5.

44. Muhsen K, Kassem E, Efraim S, Goren S, Cohen D, Ephros M. Incidence and risk factors for intussusception among children in northern Israel from 1992 to 2009: a retrospective study. BMC Pediatr. 2014;14:218.

45. Greenberg D, Givon-Lavi N, Newman N, Wheeler J, Cohen Z, Dagan R. Intussusception in children in Southern Israel: disparity between 2 populations. Pediatr Infect Dis J. 2008;27(3):236-40.

46. Yousafzai MT, Thobani R, Qazi SH, Saddal N, Yen C, Aliabadi N, et al. Intussusception among children less than 2years of age: Findings from pre-vaccine introduction surveillance in Pakistan. Vaccine. 2017.

47. Al-Malki TA. Pediatric intussusception in a Saudi Arabian tertiary hospital. West Afr J Med. 2005;24(4):309-10.

48. Crankson SJ, Al-Rabeeah AA, Fischer JD, Al-Jadaan SA, Namshan MA. Idiopathic intussusception in infancy and childhood. Saudi Med J. 2003;24 Suppl:S18-20.

49. Chouikha A, Fodha I, Maazoun K, Ben Brahim M, Hidouri S, Nouri A, et al. Rotavirus infection and intussusception in Tunisian children: implications for use of attenuated rotavirus vaccines. J Pediatr Surg. 2009;44(11):2133-8.

50. Saxena AK, Seebacher U, Bernhardt C, Hollwarth ME. Small bowel intussusceptions: issues and controversies related to pneumatic reduction and surgical approach. Acta Paediatr. 2007;96(11):1651-4.

51. Fischer TK, Bihrmann K, Perch M, Koch A, Wohlfahrt J, Kare M, et al. Intussusception in early childhood: a cohort study of 1.7 million children. Pediatrics. 2004;114(3):782-5.

52. Lappalainen S, Ylitalo S, Arola A, Halkosalo A, Rasanen S, Vesikari T. Simultaneous presence of human herpesvirus 6 and adenovirus infections in intestinal intussusception of young children. Acta Paediatr. 2012;101(6):663-70.

53. Leino T, Ollgren J, Stromberg N, Elonsalo U. Evaluation of the Intussusception Risk after Pentavalent Rotavirus Vaccination in Finnish Infants. PLoS One. 2016;11(3):e0144812.

54. Serayssol C, Abbo O, Mouttalib S, Claudet I, Labarre D, Galinier P, et al. [Seasonal pattern of intussusceptions in infants and children: is fall/winter predominance still worth consideration? A 10-year retrospective epidemiological study]. Arch Pediatr. 2014;21(5):476-82.

55. Jenke AC, Klaassen-Mielke R, Zilbauer M, Heininger U, Trampisch H, Wirth S. Intussusception: incidence and treatment-insights from the nationwide German surveillance. J Pediatr Gastroenterol Nutr. 2011;52(4):446-51.

56. Kohl LJ, Streng A, Grote V, Koletzko S, Liese JG. Intussusception-associated hospitalisations in southern Germany. Eur J Pediatr. 2010;169(12):1487-93.

57. Weiss S, Streng A, Kries R, Liese J, Wirth S, Jenke AC. Incidence of intussusception in early infancy: a capture-recapture estimate for Germany. Klin Padiatr. 2011;223(7):419-23.

58. Petursdottir K, Rosmundsson P, Hannesson PH, Moller PH. [Intussusception in children in Iceland]. Laeknabladid. 2013;99(2):77-81.

59. Eshed I, Witzling M, Gorenstein A, Serour F. [Reduction of intussusception by air enema in children--experience over a 13-year period]. Harefuah. 2003;142(10):659-61, 720, 19.

60. Costantino C, Restivo V, Cuccia M, Furnari R, Amodio E, Vitale F. Analysis of hospitalizations due to intussusception in Sicily in the pre-rotavirus vaccination era (2003-2012). Ital J Pediatr. 2015;41:52.

61. Restivo V, Costantino C, Tramuto F, Vitale F. Hospitalization rates for intussusception in children aged 0-59 months from 2009 to 2014 in Italy. Hum Vaccin Immunother. 2017;13(2):445-9.

62. Trotta F, Da Cas R, Bella A, Santuccio C, Salmaso S. Intussusception hospitalizations incidence in the pediatric population in Italy: a nationwide cross-sectional study. Ital J Pediatr. 2016;42(1):89.

63. Gadroen K, Kemmeren JM, Bruijning-Verhagen PC, Straus SM, Weibel D, de Melker HE, et al. Baseline incidence of intussusception in early childhood before rotavirus vaccine introduction, the Netherlands, January 2008 to December 2012. Euro Surveill. 2017;22(25).

64. Hilal A, MacMahon P, Cosgrove JF. Outcome of acute intussusception in a regional paediatric centre. Ir Med J. 2002;95(2):58-9.

65. Samad L, Cortina-Borja M, Bashir HE, Sutcliffe AG, Marven S, Cameron JC, et al. Intussusception incidence among infants in the UK and Republic of Ireland: a pre-rotavirus vaccine prospective surveillance study. Vaccine. 2013;31(38):4098-102.

66. Tareen F, Ryan S, Avanzini S, Pena V, Mc Laughlin D, Puri P. Does the length of the history influence the outcome of pneumatic reduction of intussusception in children? Pediatr Surg Int. 2011;27(6):587-9.

67. Tarca E, Savu B, Criscov I, Toma CM, Aprodu SG. Intussusception in infants--storm from a clear sky. Rev Med Chir Soc Med Nat Iasi. 2015;119(1):141-6.

68. Shapkina AN, Shapkin VV, Nelubov IV, Pryanishena LT. Intussusception in children: 11-year experience in Vladivostok. Pediatr Surg Int. 2006;22(11):901-4.

69. Vujovic D, Lukac M, Sretenovic A, Krstajic T, Ljubic V, Antunovic SS. Indications for repeated enema reduction of intussusception in children. Srp Arh Celok Lek. 2014;142(5-6):320-4.

70. Rubi I, Vera R, Rubi SC, Torres EE, Luna A, Arcos J, et al. Air reduction of intussusception. Eur J Pediatr Surg. 2002;12(6):387-90.

71. Buettcher M, Baer G, Bonhoeffer J, Schaad UB, Heininger U. Three-year surveillance of intussusception in children in Switzerland. Pediatrics. 2007;120(3):473-80.

72. Guney LH, Fakioglu E, Acer T, Otgun I, Arslan EE, Sagnak Akilli M, et al. Is every intussusception treatment an emergency intervention or surgery? Ulus Travma Acil Cerrahi Derg. 2016;22(2):139-44.

73. Ocal S, Cevik M, Boleken ME, Karakas E. A comparison of manual versus hydrostatic reduction in children with intussusception: single-center experience. Afr J Paediatr Surg. 2014;11(2):184-8.

74. Sonmez K, Turkyilmaz Z, Demirogullari B, Karabulut R, Kale N, Basaklar AC. Intussusception in children: experience with 105 patients in a department of paediatric surgery, Turkey. S Afr J Surg. 2012;50(2):37-9.

75. Yalcin S, Ciftci AO, Karaagaoglu E, Tanyel FC, Senocak ME. Presenting clinical features and outcome in intussusception. Indian J Pediatr. 2009;76(4):401-5.

76. Stowe J, Andrews N, Ladhani S, Miller E. The risk of intussusception following monovalent rotavirus vaccination in England: A self-controlled case-series evaluation Ref. No: JVAC-D-16-01124. Vaccine. 2016;34(50):6115.

77. Clark A, Jit M, Andrews N, Atchison C, Edmunds WJ, Sanderson C. Evaluating the potential risks and benefits of infant rotavirus vaccination in England. Vaccine. 2014;32(29):3604-10.

78. Samad L, Marven S, El Bashir H, Sutcliffe AG, Cameron JC, Lynn R, et al. Prospective surveillance study of the management of intussusception in UK and Irish infants. Br J Surg. 2012;99(3):411-5.

79. Latipov R, Khudoyorov R, Flem E. Childhood intussusception in Uzbekistan: analysis of retrospective surveillance data. BMC Pediatr. 2011;11:22.

80. Zaman K, Breiman RF, Yunus M, Arifeen SE, Mahmud A, Chowdhury HR, et al. Intussusception surveillance in a rural demographic surveillance area in bangladesh. J Infect Dis. 2009;200 Suppl 1:S271-6.

81. Satter SM, Aliabadi N, Yen C, Gastanaduy PA, Ahmed M, Mamun A, et al. Epidemiology of childhood intussusception in Bangladesh: Findings from an active national hospital based surveillance system, 2012-2016. Vaccine. 2017.

82. Bahl R, Saxena M, Bhandari N, Taneja S, Mathur M, Parashar UD, et al. Population-based incidence of intussusception and a case-control study to examine the association of intussusception with natural rotavirus infection among indian children. J Infect Dis. 2009;200 Suppl 1:S277-81.

83. Bhowmick K, Kang G, Bose A, Chacko J, Boudville I, Datta SK, et al. Retrospective surveillance for intussusception in children aged less than five years in a South Indian tertiary-care hospital. J Health Popul Nutr. 2009;27(5):660-5.

84. Gupta M, Kanojia R, Singha R, Tripathy JP, Mahajan K, Saxena A, et al. Intussusception Rate Aamong Uunder-Five-Children Before Introduction of Rotavirus Vaccine in North India. J Trop Pediatr. 2017.

85. Jehangir S, John J, Rajkumar S, Mani B, Srinivasan R, Kang G. Intussusception in southern India: comparison of retrospective analysis and active surveillance. Vaccine. 2014;32 Suppl 1:A99-103.

86. John J, Kawade A, Rongsen-Chandola T, Bavdekar A, Bhandari N, Taneja S, et al. Active surveillance for intussusception in a phase III efficacy trial of an oral monovalent rotavirus vaccine in India. Vaccine. 2014;32 Suppl 1:A104-9.

87. Mathew MA, Venugopal S, Arora R, Kang G. Leveraging the National Rotavirus Surveillance Network for Monitoring Intussusception. Indian Pediatr. 2016;53(7):635-8.

88. Mehendale S, Kumar CP, Venkatasubramanian S, Prasanna T. Intussusception in Children Aged Less than Five years. Indian J Pediatr. 2016;83(10):1087-92.

89. Ramachandran P, Gupta A, Vincent P, Sridharan S. Air enema for intussusception: is predicting the outcome important? Pediatr Surg Int. 2008;24(3):311-3.

90. Raman T, Mukhopadhyaya A, Eapen CE, Aruldas V, Bose A, Sen S, et al. Intussusception in southern Indian children: lack of association with diarrheal disease and oral polio vaccine immunization. Indian J Gastroenterol. 2003;22(3):82-4.

91. Singh JV, Kamath V, Shetty R, Kumar V, Prasad R, Saluja T, et al. Retrospective surveillance for intussusception in children aged less than five years at two tertiary care centers in India. Vaccine. 2014;32 Suppl 1:A95-8.

92. Srinivasan R, Girish Kumar CP, Naaraayan SA, Jehangir S, Thangaraj JWV, Venkatasubramanian S, et al. Intussusception hospitalizations before rotavirus vaccine introduction: Retrospective data from two referral hospitals in Tamil Nadu, India. Vaccine. 2017.

93. Rayamajhi A, Thapa A, Kumar M, Yen C, Tate JE, Parashar UD, et al. Preparing for rotavirus vaccine introduction - A retrospective assessment of the epidemiology of intussusception in children below 2years of age in Nepal. Vaccine. 2017.

94. Shakya VC, Agrawal CS, Sinha AK, Bhatta NK, Khania S, Adhikary S. Childhood Intussusception: A Prospective Institutional Study at BPKIHS. Available at: <https://www.nepjol.info/index.php/JNPS/article/view/3862/3519> [accessed 28th July 2018]. J Nepal Paediatr Soc. 2011;31(1).

95. Thapa B, Chaudhary RP, Pun MS, Ral GK. Clinical Analysis, Management and Outcome of Intussusception in Children. Journal of Nepal Paediatric Society. 2012;32(1).

96. Kruatrachue A, Wongtapradit L, Nithipanya N, Ratanaprakarn W. Result of air enema reduction in 737 cases of intussusception. J Med Assoc Thai. 2011;94 Suppl 3:S22-6.

97. Khumjui C, Doung-ngern P, Sermgew T, Smitsuwan P, Jiraphongsa C. Incidence of intussusception among children 0-5 years of age in Thailand, 2001-2006. Vaccine. 2009;27 Suppl 5:F116-9.

98. Pruksananonda P, Athirakul K, Worawattanakul M, Varavithya W, Pisithpun A, Kitayaporn D, et al. Intussusception in a private tertiary-care hospital, Bangkok, Thailand: a case series. Southeast Asian J Trop Med Public Health. 2007;38(2):339-42.

99. Blanch AJ, Perel SB, Acworth JP. Paediatric intussusception: epidemiology and outcome. Emerg Med Australas. 2007;19(1):45-50.

100. Justice F, Carlin J, Bines J. Changing epidemiology of intussusception in Australia. J Paediatr Child Health. 2005;41(9-10):475-8.

101. Justice FA, Auldist AW, Bines JE. Intussusception: trends in clinical presentation and management. J Gastroenterol Hepatol. 2006;21(5):842-6.

102. Lloyd-Johnsen C, Justice F, Donath S, Bines JE. Retrospective hospital based surveillance of intussusception in children in a sentinel paediatric hospital: benefits and pitfalls for use in post-marketing surveillance of rotavirus vaccines. Vaccine. 2012;30 Suppl 1:A190-5.

103. Palupi-Baroto R, Lee KJ, Carlin JB, Bines JE. Intussusception in Australia: epidemiology prior to the introduction of rotavirus vaccine. Aust N Z J Public Health. 2015;39(1):11-4.

104. Cui P, Liu N, Li J, Huang T, Ge H, Wu Q, et al. [Epidemiology of intussusception related hospitalizations in children aged <2 years in Suzhou, 2007-2013]. Zhonghua Liu Xing Bing Xue Za Zhi. 2016;37(3):410-4.

105. Cui LL, Geng XY, Zhang J, Zhang J. [Epidemiological characteristics and risk factors of primary intussusception in children among two years old and below, Ji'nan city]. Zhonghua Yu Fang Yi Xue Za Zhi. 2018;52(7):727-33.

106. Liu N, Yen C, Huang T, Cui P, Tate JE, Jiang B, et al. Incidence and epidemiology of intussusception among children under 2years of age in Chenzhou and Kaifeng, China, 2009-2013. Vaccine. 2018.

107. Zhang Y, Bai YZ, Li SX, Liu SJ, Ren WD, Zheng LQ. Sonographic findings predictive of the need for surgical management in pediatric patients with small bowel intussusceptions. Langenbecks Arch Surg. 2011;396(7):1035-40.

108. Hong Kong Intussusception Study G. Intussusception trends in Hong Kong children. Hong Kong Med J. 2007;13(4):279-83.

109. Wong CW, Chan IH, Chung PH, Lan LC, Lam WW, Wong KK, et al. Childhood intussusception: 17-year experience at a tertiary referral centre in Hong Kong. Hong Kong Med J. 2015;21(6):518-23.

110. Nakagomi T, Takahashi Y, Arisawa K, Nakagomi O. A high incidence of intussusception in Japan as studied in a sentinel hospital over a 25-year period (1978-2002). Epidemiol Infect. 2006;134(1):57-61.

111. Noguchi A, Nakagomi T, Kimura S, Takahashi Y, Matsuno K, Koizumi H, et al. Incidence of intussusception as studied from a hospital-based retrospective survey over a 10-year period (2001-2010) in Akita Prefecture, Japan. Jpn J Infect Dis. 2012;65(4):301-5.

112. Takeuchi M, Osamura T, Yasunaga H, Horiguchi H, Hashimoto H, Matsuda S. Intussusception among Japanese children: an epidemiologic study using an administrative database. BMC Pediatr. 2012;12:36.

113. Giak CL, Singh HS, Nallusamy R, Leong TY, Ng TL, Bock HL. Epidemiology of intussusception in Malaysia: a three-year review. Southeast Asian J Trop Med Public Health. 2008;39(5):848-55.

114. Chen YE, Beasley S, Grimwood K, New Zealand Rotavirus Study G. Intussusception and rotavirus associated hospitalisation in New Zealand. Arch Dis Child. 2005;90(10):1077-81.

115. Kodikara H, Lynch A, Morreau P, Vogel S. Ten-year review of intussusception at Starship Hospital: 1998-2007. N Z Med J. 2010;123(1324):32-40.

116. Phua KB, Lee BW, Quak SH, Jacobsen A, Teo H, Vadivelu-Pechai K, et al. Incidence of intussusception in Singaporean children aged less than 2 years: a hospital-based prospective study. BMC Pediatr. 2013;13:161.

117. Tan N, Teoh YL, Phua KB, Quak SH, Lee BW, Teo HJ, et al. An update of paediatric intussusception incidence in Singapore: 1997-2007, 11 years of intussusception surveillance. Ann Acad Med Singapore. 2009;38(8):690-2.

118. Yap Shiyi E, Ganapathy S. Intussusception in Children Presenting to the Emergency Department: An Asian Perspective. Pediatr Emerg Care. 2017;33(6):409-13.

119. Jo DS, Nyambat B, Kim JS, Jang YT, Ng TL, Bock HL, et al. Population-based incidence and burden of childhood intussusception in Jeonbuk Province, South Korea. Int J Infect Dis. 2009;13(6):e383-8.

120. Chen SC, Wang JD, Hsu HY, Leong MM, Tok TS, Chin YY. Epidemiology of childhood intussusception and determinants of recurrence and operation: analysis of national health insurance data between 1998 and 2007 in Taiwan. Pediatr Neonatol. 2010;51(5):285-91.

121. Hsiao CC, Tsao LY, Lai CH. Nationwide population-based epidemiologic study of childhood and adulthood intussusception in Taiwan. Pediatr Neonatol. 2013;54(3):188-93.

122. Hsu WL, Lee HC, Yeung CY, Chan WT, Jiang CB, Sheu JC, et al. Recurrent Intussusception: when Should Surgical Intervention be performed? Pediatr Neonatol. 2012;53(5):300-3.

123. Yen C, Shih SM, Tate JE, Wu FT, Huang YC, Parashar UD, et al. Intussusception-related Hospitalizations Among Infants Before and After Private Market Licensure of Rotavirus Vaccines in Taiwan, 2001-2013. Pediatr Infect Dis J. 2017;36(10):e252-e7.

124. Bines JE, Liem NT, Justice FA, Son TN, Kirkwood CD, de Campo M, et al. Risk factors for intussusception in infants in Vietnam and Australia: adenovirus implicated, but not rotavirus. J Pediatr. 2006;149(4):452-60.

125. Trang NV, Burnett E, Ly LH, Anh NP, Hung PH, Linh HM, et al. Recurrent intussusception among infants less than 2 years of age in Vietnam. Vaccine. 2018.

126. Van Trang N, Le Nguyen NT, Dao HT, Ho VL, Tran DT, Loewen J, et al. Incidence and Epidemiology of Intussusception among Infants in Ho Chi Minh City, Vietnam. J Pediatr. 2014;164(2):366-71.

127. Tran LA, Yoshida LM, Nakagomi T, Gauchan P, Ariyoshi K, Anh DD, et al. A High Incidence of Intussusception Revealed by a Retrospective Hospital-Based Study in Nha Trang, Vietnam between 2009 and 2011. Trop Med Health. 2013;41(3):121-7.
